# Supplementary material for: AAVR disruption as a producer-cell engineering strategy for enhanced supernatant-based recovery of selected AAV serotypes
Source: Mol Ther Adv. 2026 Aug 3;34(3):201830. doi: 10.1016/j.omta.2026.201830 (PMC13519645; doi:10.1016/j.omta.2026.201830)
Supplement: Supplementary file 1 [file mmc1.pdf]

OMTA, Volume *34*

Supplemental information

AAVR disruption as a producer-cell engineering strategy for enhanced supernatant-based recovery of selected AAV serotypes

Kenji Sakamoto, Kouhei Yoshida, Yuji Tsunekawa, Naymel A. Guzman Mendoza, Yui Komaki, Azusa Onodera, and Takashi Okada

A

	Position	Targeting sequence (coding KIAA0319L)
gRNA 1	Chromosome 1: 35,555,095 - 35,555,120	ATTCAACAGATACTTACGTC AGG
gRNA 2	Chromosome 1: 35,553,781 - 35,553,806	CCTGGCAAGAGGCTTTACCC TGG

Genotyping primer	Position	Sequence
Amp F primer	Chromosome 1: 35,553,450 – 35,553,479 (+)	AACCCCAAAGCTGATGTTACTGAAATTGCT
Amp R primer	Chromosome 1: 35,556,038 – 35,556,067 (-)	TGGTGATGTGTTGTTGTTCTTGCGTTTGGT

B

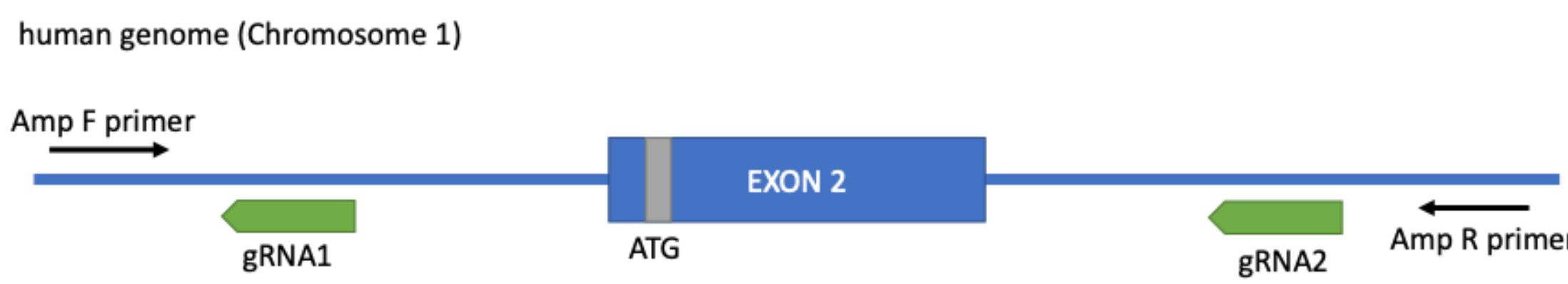

Figure S1: CRISPR/Cas9 targeting strategy for AAVR (KIAA0319L) knockout
(A) gRNA and genotyping primer sequences. Genomic target sites for CRISPR/Cas9-mediated disruption of AAVR (KIAA0319L) are shown. Two guide RNAs (gRNA1 and gRNA2) flanking exon 2 were designed, and their genomic positions and targeting sequences are listed. Genotyping primers flanking the targeted region were used to detect exon 2 deletion; their genomic positions and sequences are provided.
(B) Schematic illustration of the AAVR exon 2 targeting strategy. The positions of gRNA target sites and genotyping primers relative to exon 2 are illustrated.

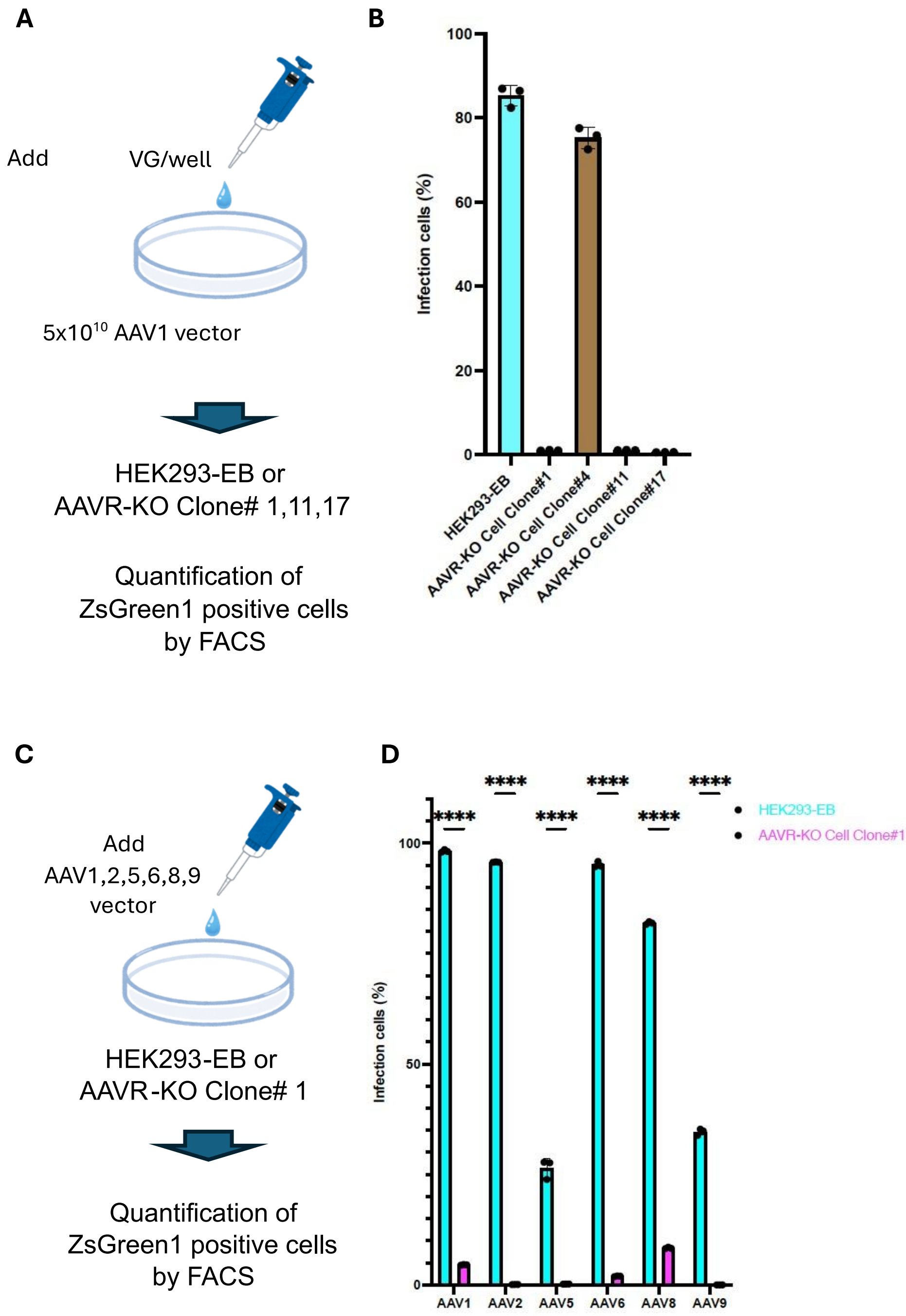

Figure S2: Quantitative analysis of serotype-dependent AAV transduction in AAVR-knockout cells.
(A) Schematic illustration of experimental design for assessing transduction efficiency of AAV1 across parental HEK293-EB and independent AAVR-KO clones (#1, #11, #17).
(B) Transduction efficiency of AAV1 across parental HEK293-EB and AAVR-KO clones (#1, #11, #17), quantified by ZsGreen1 expression. Data represent mean ± SD. Data are presented as mean ± SD (n = 3 technical replicates within a single experiment).

(C) Schematic illustration of experimental design for assessing transduction efficiency of serotype-dependent transduction efficiency (AAV1, 2, 5, 6, 8, 9), corresponding to Fig. 1C.

(D) Quantification of serotype-dependent transduction efficiency corresponding to Fig. 1C. Parental HEK293-EB cells and AAVR-KO clone #1 cells were transduced with ZsGreen1-expressing AAV serotypes 1, 2, 5, 6, 8, and 9. Transduction efficiency was quantified based on the percentage of ZsGreen1-positive cells. The input doses were as follows: AAV1: 6.1×10^4 VG/cell; AAV2: 1.9×10^4 VG/cell; AAV5: 5.2×10^4 VG/cell; AAV6: 1.5×10^4 VG/cell; AAV8: 1.2×10^5 VG/cell; and AAV9: 2.9×10^4 VG/cell. Data are presented as mean ± SD (n = 3 technical replicates from a single experiment). Statistical analysis was performed using two-way ANOVA followed by Sidak's multiple comparisons test. ****$P < 0.0001$.

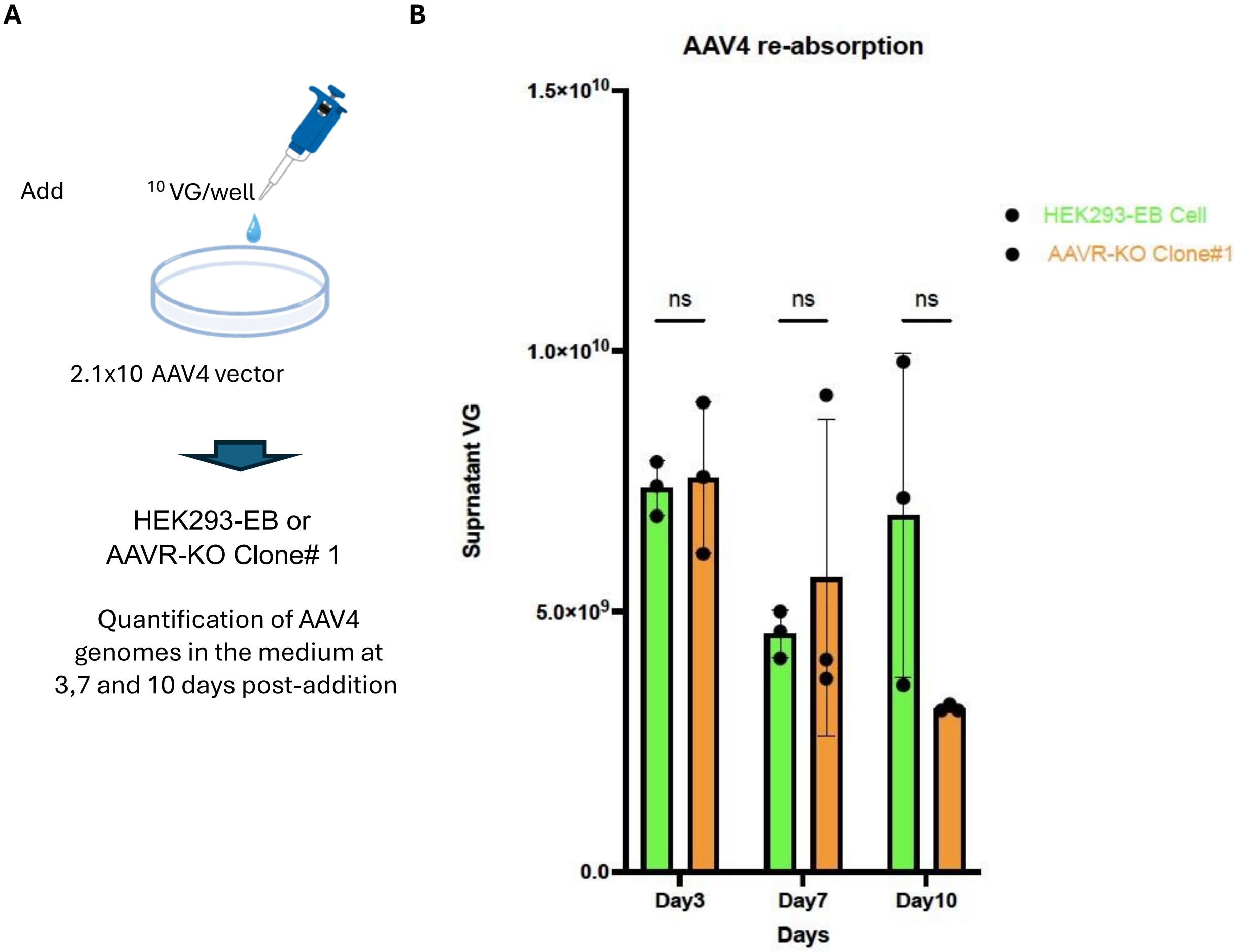

Figure S3: Cellular uptake of extracellular AAV4 particles in parental and AAVR-knockout cells.

(A) Schematic illustration of experimental design. Purified AAV4 vector (2.1×10^{10} VG per well; 1.05×10^{10} VG/mL) was added to subconfluent parental HEK293-EB or AAVR-KO clone #1 cells cultured in 6-well plates (2 mL medium per well). Vector genomes remaining in the culture supernatant were quantified at 3, 7 and 10 days post-addition.

(B) Quantification of AAV4 genomes in the supernatant. AAV4 showed no significant difference in supernatant vector genome levels between parental and AAVR-KO cells over time. Data represent mean ± SD (n = 3 technical replicates within a single experiment). Statistical analysis was performed using two -way ANOVA with Sidak's multiple comparisons test.

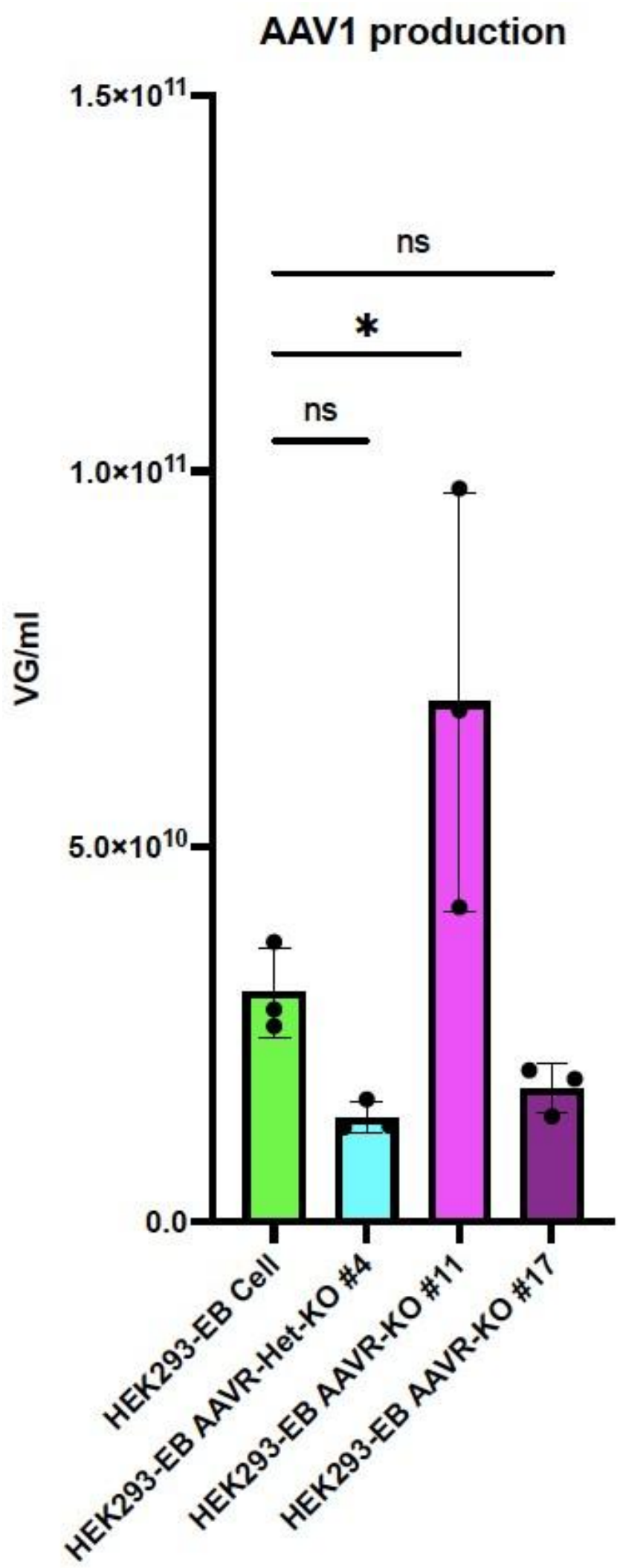

Figure S4: AAV1 extracellular recovery in AAVR-KO and HEK293-EB clones.
Supernatant VG titers following extracellular recovery of AAV1 in parental HEK293-EB or AAVR-KO clone #11, 17 and Het-KO clone#4 cells. AAVR knockout clone#11 showed significantly increased extracellular titers of AAV1but AAVR-KO clone #17 and Het-KO clone#4 showed no significant change. Data are presented as men ± SD (n = 3 technical replicates within a single experiment). Statistical analysis was performed using ordinary one-way ANOVA. *P < 0.05.

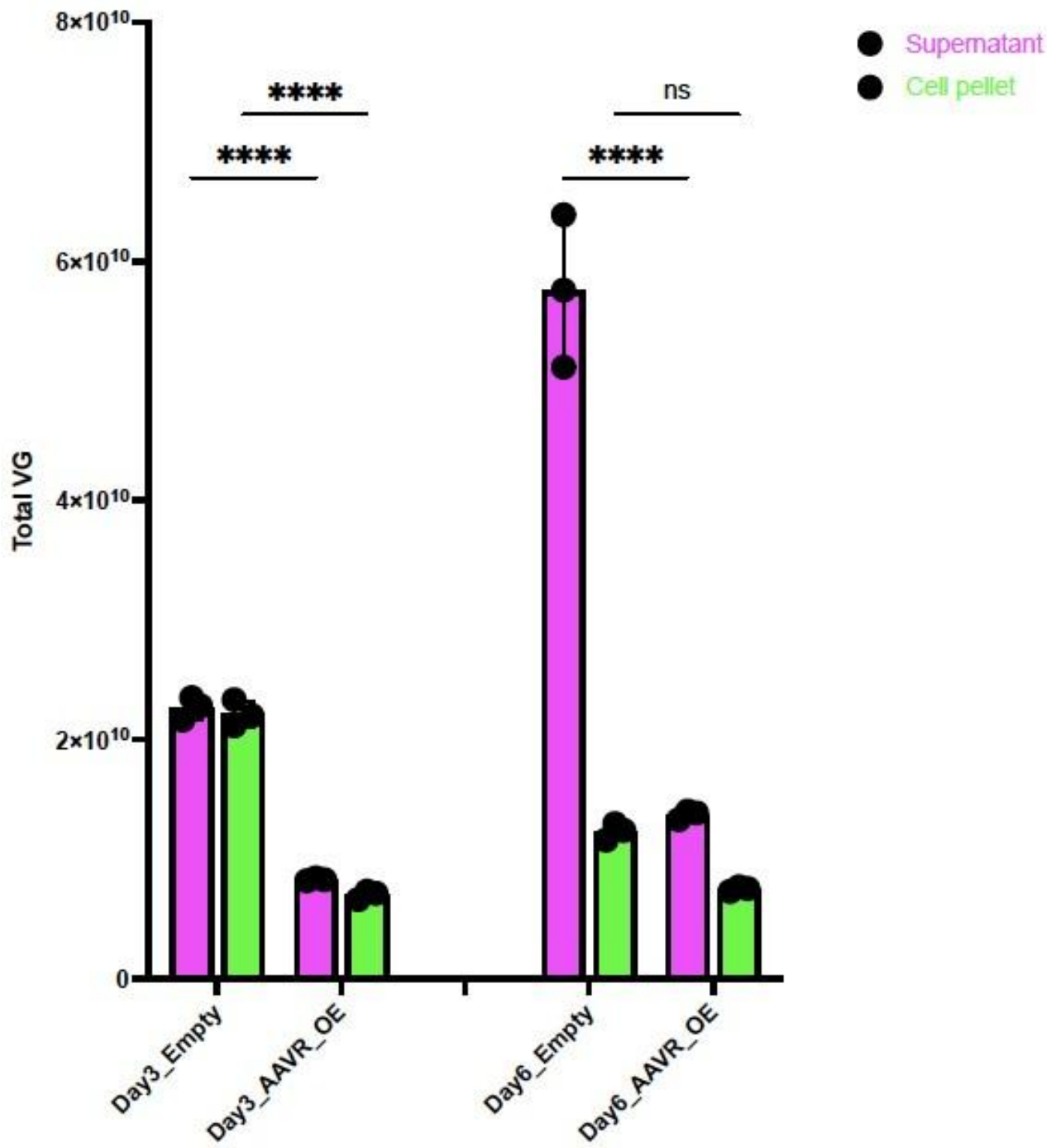

Figure S5: AAVR overexpression alters supernatant and cell-pellet AAV1 vector genome levels during production.
HEK293-EB cells were transfected with pR2C1, pHelper, pAAV-ZsGreen1, and either the pCAX empty vector backbone (Empty condition) or pCAX-CAG-AAVR (AAVR-OE condition) at a 1:1:1:1 molar ratio. AAV1 vector genomes yield from culture supernatant and cell pellet were quantified at 3 and 6 days post-transfection. AAVR overexpression reduced the accumulation of extracellular vector genomes from day 3 to day 6, consistent with the involvement of AAVR expression levels in supernatant vector recovery. AAVR overexpression also reduced cell-associated vector genomes at day 3, suggesting that AAVR may influence vector production or intracellular vector handling in addition to extracellular particle re-uptake. Data represent mean ± SD. Data are presented as mean ± SD (n = 3 technical replicates within a single experiment). Statistical analysis was performed on data using two-way ANOVA with Sidak's multiple comparisons test. ****P < 0.0001.

Table S1. Rate of supernatant AAV1 vector genome loss in parental and AAVR-knockout HEK293-EB cells.

The rate of decline in supernatant AAV1 vector genome levels was calculated using the defined input dose as day 0. Slopes were estimated by linear regression of log10-transformed supernatant vector genome levels from day 0 to day 9.

Cell line	Day0	Day3 mean	Day6 mean	Day9 mean	Linear slope, VG/day	log10 slope/day	Day9/Day0 remaining
HEK293-EB	5.00 × 10^10	1.67 × 10^10	1.09 × 10^10	5.65 × 10^9	-4.44 * 10^9/DAY	-0.1008/DAY	11.30%
AAVR-KO #1	5.00 × 10^10	2.32 × 10^10	2.05 × 10^10	1.42 × 10^10	-3.62 * 10^9/DAY	-0.0564/DAY	28.40%
AAVR-KO #11	5.00 × 10^10	2.34 × 10^10	1.91 × 10^10	1.48 × 10^10	-3.62 * 10^9/DAY	-0.0558/DAY	29.60%
AAVR-KO #17	5.00 × 10^10	2.49 × 10^10	2.28 × 10^10	1.95 × 10^10	-3.03 * 10^9/DAY	-0.0422/DAY	39.00%

Table S2. Supernatant vector genome titers of AAV serotypes produced in parental and AAVR-knockout HEK293-EB cells.

Supernatant vector genome titers of AAV1, AAV2, AAV5, AAV6, AAV8, and AAV9 produced in parental HEK293-EB cells and AAVR-KO clone #1 cells are shown. Values are presented as log10-transformed vector genome titers. Mean differences, 95% confidence intervals, fold changes, and adjusted P values were calculated from log10-transformed data using two-way ANOVA followed by Sidak's multiple comparisons test.

Serotype	Mean log10 VG titer in parental HEK293-EB cells	Mean log10 VG titer in AAVR-KO cells	Difference, AAVR-KO − parental (log10 units)	95% CI of difference	Approximate fold change	Adjusted P value	Interpretation
AAV1	10.31	11	0.6884	0.2526 to 1.124	4.9-fold increase	0.0008	Significant increase
AAV2	9.785	10.1	0.311	−0.1248 to 0.7468	2.0-fold increase	0.2738	Not significant
AAV5	10.87	10.83	−0.04888	−0.4847 to 0.3869	0.89-fold	0.9998	Not significant
AAV6	9.655	10.64	0.9875	0.5517 to 1.423	9.7-fold increase	<0.0001	Significant increase
AAV8	10.74	11.22	0.4786	0.04278 to 0.9143	3.0-fold increase	0.0259	Significant increase
AAV9	11.35	11.31	−0.03326	−0.4690 to 0.4025	0.93-fold	>0.9999	Not significant

Supplemental Materials and Methods

AAVR knock-out cell generation

AAVR-knockout HEK293-EB clones were generated using a CRISPR/Cas9-mediated genome editing strategy. Subconfluent HEK293-EB cells were transfected with pCAX-SpCas9 and two gRNA plasmids targeting sequences flanking exon 2 of KIAA0319L using PEI-Max (Polysciences). Five days after transfection, single cells were sorted into 96-well plates using a BD FACS Melody cell sorter. After approximately 2 weeks of culture, colonies were expanded and screened by genomic PCR to identify clones carrying homozygous deletion of exon 2. Loss of AAVR protein expression was further confirmed by Western blotting.

Genotyping PCR

Genomic DNA was extracted from expanded clones using the DNeasy Blood & Tissue Kit (QIAGEN) according to the manufacturer's instructions. PCR-based genotyping was performed using PrimeSTAR GXL DNA Polymerase (Takara Bio). Each 50-µL PCR reaction contained 10 µL of 5× PrimeSTAR GXL buffer, 4 µL of dNTP mixture, 1 µL of PrimeSTAR GXL DNA Polymerase, 0.5 µL each of forward and reverse primers (10 pM), 5 µL of betaine, 1 µL of genomic DNA template (50 ng), and 28 µL of distilled water. PCR was performed using the following cycling conditions and Primer: initial denaturation at 96°C for 5 min, followed by 33 cycles of denaturation at 96°C for 20 s and extension at 68°C for 2 min (F-Primer: AACCCCAAAGCTGATGTTACTGAAATTGCT, R-Primer: TGGTGATGTGTTGTTGTTCTTGCGTTTGGT). PCR products were analyzed by electrophoresis on a 1% agarose gel, image was taken by ChemiDoc imaging system (BioRad).

AAVR overexpression production assay

HEK293-EB cells were seeded in 6-well culture plates at a density of 4×10^4 cells/cm² and cultured for 3 days at 37°C in a 5% CO_2 atmosphere until reaching approximately 90% confluence. Cells were then transfected with pR2C1, pHelper, pAAV-ZsGreen1, and either the pCAX empty vector backbone or pCAX-CAG-AAVR using PEI Max. The four plasmids were transfected at a 1:1:1:1 molar ratio. After transfection, cells were maintained in serum-free medium without medium exchange. Culture supernatants and cells were collected at 3 and 6 days post-transfection. Supernatants were clarified by

centrifugation, and cells were harvested separately as the cell-associated fraction. The harvested cells were lysed with 0.1% Triton X-100 for 2 h. Vector genomes in the culture supernatant and cell-associated fractions were quantified by qPCR.
