# Supplementary material for: AAVR disruption as a producer-cell engineering strategy for enhanced supernatant-based recovery of selected AAV serotypes
Source: Mol Ther Adv. 2026 Aug 3;34(3):201830. doi: 10.1016/j.omta.2026.201830 (PMC13519645; doi:10.1016/j.omta.2026.201830)
Supplement: Supplementary file 2 [file mmc2.pdf]

Molecular Therapy
Advances
Brief report

AAVR disruption as a producer-cell engineering strategy for enhanced supernatant-based recovery of selected AAV serotypes

Kenji Sakamoto,[1,3] Kouhei Yoshida,[1,2,3] Yuji Tsunekawa,[1,3] Naymel A. Guzman Mendoza,[1] Yui Komaki,[1] Azusa Onodera,[1] and Takashi Okada[1]

[1]Division of Molecular and Medical Genetics, Center for Gene and Cell Therapy, The Institute of Medical Science, The University of Tokyo, Tokyo, Japan; [2]Tosoh Corporation, Life Science Research Laboratory, Ayase, Kanagawa 252-1123, Japan

Recovery of adeno-associated virus (AAV) vectors from culture supernatants has attracted increasing interest as a strategy to simplify downstream processing and improve product purity. However, several AAV serotypes can be re-internalized by producer cells, raising the possibility that newly released particles are recaptured during production and thereby limiting extracellular yield. Because AAV entry is mediated by the universal receptor KIAA0319L (AAVR), receptor-dependent re-uptake may contribute to this limitation, yet its role in vector manufacturing remains unexplored. Here, we investigated whether disruption of AAVR enhances supernatant-based AAV recovery. Using CRISPR-Cas9-mediated genome editing, we generated AAVR-knockout (AAVR-KO) HEK293-EB producer cells by deleting exon 2 containing the translational start codon. AAVR ablation abolished susceptibility to multiple AAV serotypes and markedly reduced cellular uptake of extracellular particles. In supernatant AAV vector recovery assays, AAVR knockout increased supernatant vector genome recovery for selected serotypes in a representative clone, although this effect varied among independently isolated clones. Complementary AAVR overexpression reduced accumulation of extracellular AAV1 vector genomes, supporting a role for AAVR expression levels in supernatant recovery while also suggesting additional effects on production-related cellular processes. These findings identify AAVR-dependent cellular uptake or retention as a modifiable post-release process that can influence supernatant-based AAV vector recovery.

INTRODUCTION

Recent advances in gene therapy have positioned adeno-associated virus (AAV) vectors as a leading platform for therapeutic gene delivery due to their broad tropism, long-term expression, and favorable safety profile.[1,2] AAV vectors are commonly produced in HEK293 cells by triple plasmid transfection. While vectors were traditionally recovered from cell lysates, most serotypes, except AAV2, are efficiently released into the culture supernatant under serum-free conditions.[3] Supernatant-based recovery can reduce the burden of process-related impurities (e.g., cell debris, host-cell proteins, and host-cell DNA) and streamline downstream processing, making it an increasingly attractive option for research and, in some settings, scalable manufacturing.[4–9]

Vector accumulation in the supernatant typically peaks 5–7 days post-transfection.[3] However, several serotypes (e.g., AAV1, AAV2, and AAV6) efficiently infect HEK293-based producer cell lines, raising the possibility that newly released particles may be reabsorbed and degraded. Although different serotypes utilize distinct entry factors, the KIAA0319L gene product, designated the universal AAV receptor (AAVR), has been identified as a critical mediator of infection across multiple capsids.[10] We therefore hypothesized that AAVR may paradoxically limit vector recovery during production by mediating re-uptake of extracellular AAV particles. If so, genetic ablation of AAVR in producer cells should prevent vector re-absorption and enhance extracellular vector accumulation.

RESULTS

To test this hypothesis, we generated AAVR-knockout (AAVR-KO) HEK293-EB producer cell lines. To genetically ablate AAVR, we engineered HEK293-EB producer cells[11] using a CRISPR-Cas9 strategy designed to generate a functional null allele. Two guide RNAs (gRNA) were designed to flank exon 2, which contains the translational start codon of AAVR, thereby enabling excision of the entire exon upon Cas9-mediated double-strand cleavage (Figure S1). Cells were transfected with SpCas9 and the paired gRNA expression constructs, and clonal populations were isolated (total 32 clones).

Received 16 March 2026; accepted 30 July 2026;
https://doi.org/10.1016/j.omta.2026.201830.

[3]These authors contributed equally

Correspondence: Yuji Tsunekawa, Division of Molecular and Medical Genetics, Center for Gene and Cell Therapy, The Institute of Medical Science, The University of Tokyo, Tokyo, Japan.
E-mail: yuji-tsunekawa@g.ecc.u-tokyo.ac.jp
Correspondence: Takashi Okada, Division of Molecular and Medical Genetics, Center for Gene and Cell Therapy, The Institute of Medical Science, The University of Tokyo, Tokyo, Japan.
E-mail: t-okada@ims.u-tokyo.ac.jp

Published by Elsevier Inc. on behalf of The American Society of Gene and Cell Therapy.
This is an open access article under the CC BY license (http://creativecommons.org/licenses/by/4.0/).

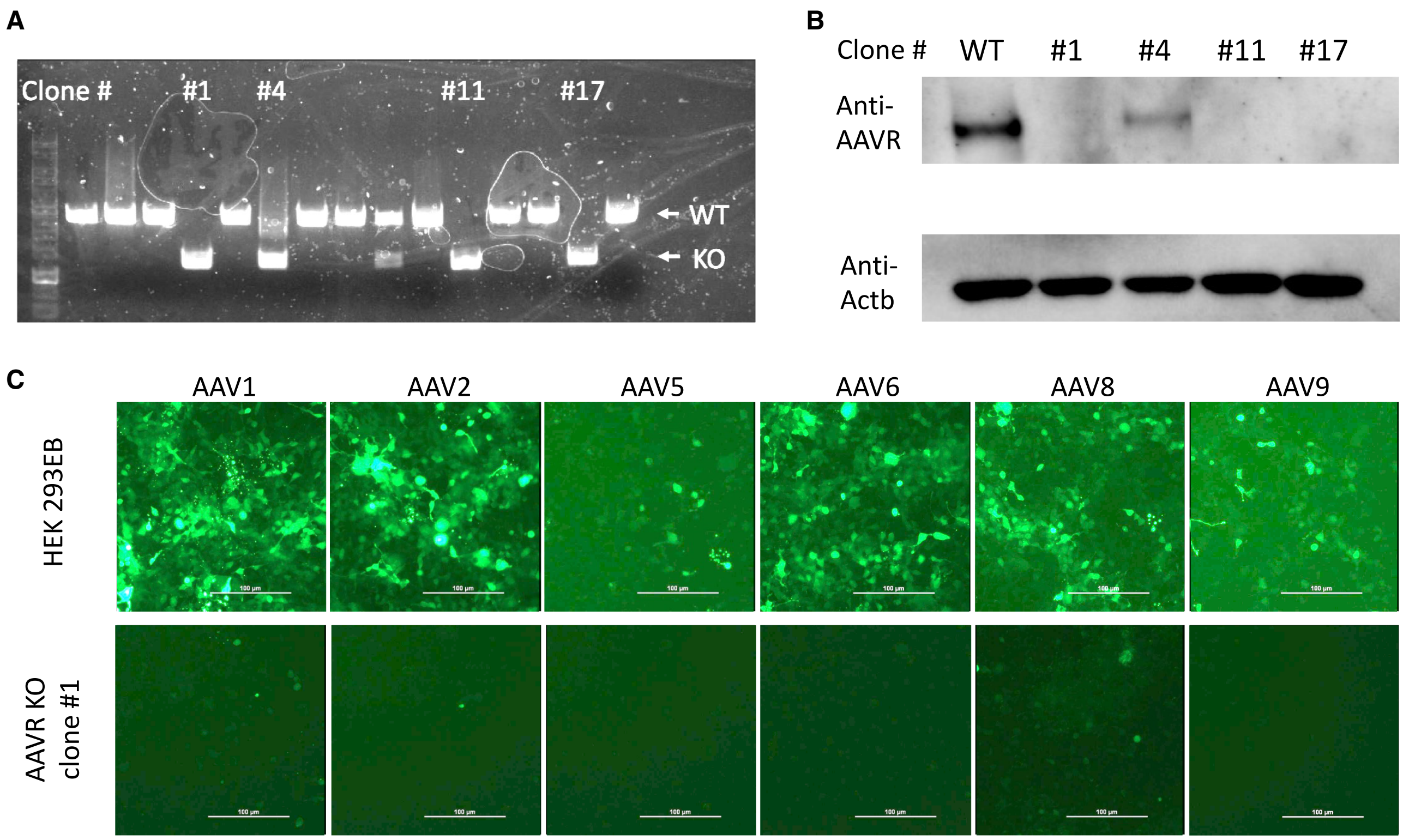

Figure 1. Generation and functional validation of AAVR-knockout producer cells

(A) PCR genotyping of AAVR-knockout clones. Deletion of exon 2 of AAVR generated a shorter PCR fragment compared with the wild-type allele, confirming successful knockout in representative clones (#1, #11, and #17).

(B) Immunoblot confirmation of AAVR knockout. Western blot analysis demonstrated complete loss of AAVR protein in selected clones, while clone #4 exhibited reduced expression consistent with a heterozygous genotype. β-Actin was used as a loading control.

(C) Functional validation of AAVR ablation. Parental HEK293-EB cells and AAVR-KO clone #1 were transduced with ZsGreen1-expressing AAV serotypes 1, 2, 5, 6, 8, and 9. Transduction efficiency was assessed by ZsGreen1 fluorescence. AAVR knockout markedly reduced susceptibility to AAV1, AAV2, AAV5, AAV6, AAV8, and AAV9. Scale bars, 100 μm.

Deletion of exon 2 was confirmed by genomic PCR-based genotyping, and loss of AAVR protein expression was verified by immunoblot analysis. Multiple independent knockout clones (#1, #11, and #17) exhibited complete absence of detectable AAVR protein, whereas clone #4 showed reduced but residual AAVR expression consistent with a heterozygous knockout genotype (Figures 1A and 1B). To functionally validate receptor ablation, we first assessed AAV1 transduction across all independent AAVR-KO clones. While parental HEK293-EB cells were efficiently transduced, clones #1, #11, and #17 uniformly exhibited almost complete loss of susceptibility to AAV1-mediated transduction (Figures S2A and S2B), confirming functional disruption of AAVR-dependent productive transduction. Having established concordant phenotypes across independent clones, we selected clone #1 as a representative AAVR-null producer cell line for subsequent analyses. We then evaluated susceptibility across multiple serotypes (AAV1, 2, 5, 6, 8, and 9) using ZsGreen1-expressing vectors.[11] Transduction was markedly reduced for all serotypes tested, although the magnitude of impairment varied (Figure 1C; Figures S2C and S2D), consistent with differential dependence of cell transduction on AAVR among capsids[10]

We next tested whether AAVR contributes to the loss or cellular retention of extracellular AAV particles using exogenously added purified AAV1 vector. Purified AAV1 vector (5×10^{10} VG/well) was added to subconfluent HEK293-EB or AAVR-KO cells, and vector genome (VG) remaining in the culture supernatant were quantified at 3, 6, and 9 days post-addition (Figure 2A). The day 3, 6, and 9 time points were selected to capture early and late changes in extracellular AAV1 retention while avoiding potential confounding effects associated with prolonged culture without medium exchange. In HEK293-EB cells, supernatant VG levels declined substantially over time, indicating progressive removal of extracellular particles. In contrast, AAVR-KO clones retained significantly higher VG levels at all three time points (Figure 2B). To further assess whether the decline could be explained by uniform extracellular degradation, we calculated the rate of VG loss using the defined input dose as day 0. Linear regression of log10-transformed supernatant AAV1 VG levels from day 0 to day 9 showed a steeper decline in parental HEK293-EB cells (−0.1008 log10 VG/day) than in AAVR-KO clones #1, #11, and #17 (−0.0564, −0.0558, and −0.0422 log10 VG/day, respectively) (Table S1). These results argue against uniform

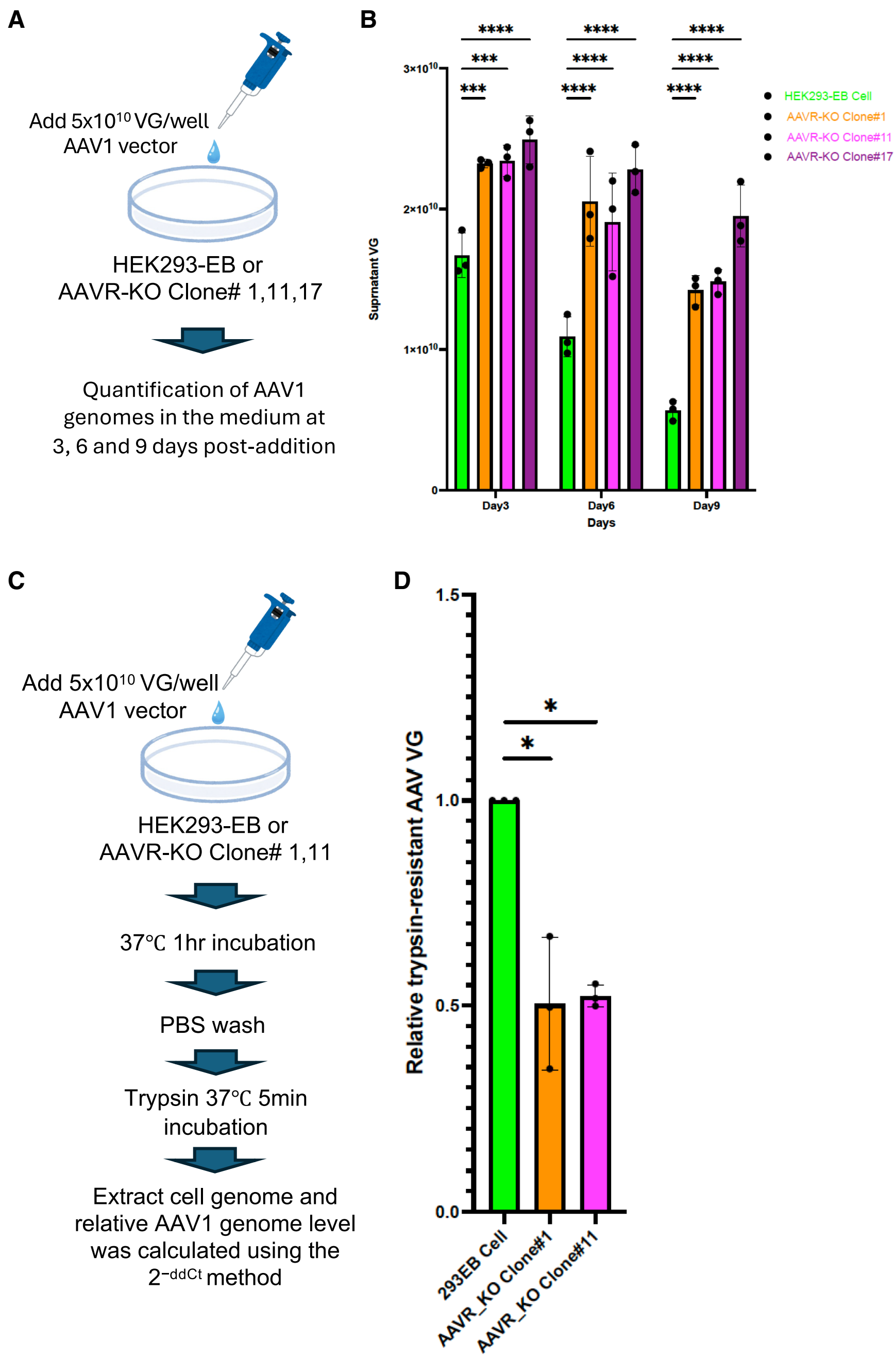

Figure 2. AAVR mediates medium retention of extracellular AAV particles

(A) Schematic illustration of experimental design for assessing AAV vector medium retention. Purified AAV1 vector (5×10^{10} VG per well; 2.5×10^{10} VG/mL) was added to subconfluent parental HEK293-EB or AAVR-KO clone #1, #11, and #17 cells cultured in 6-well plates (2 mL medium per well). Vector genomes remaining in the culture supernatant were quantified at 3, 6, and 9 days post-addition.

(B) Quantification of AAV1 genomes in the supernatant. Parental HEK293-EB cells showed progressive decline in extracellular VG levels, whereas AAVR-KO cells retained significantly higher VG levels at the indicated time points. Data represent mean ± SD (n = 3 technical replicates within a single experiment). Statistical analysis was performed on data using two-way ANOVA with Sidak's multiple comparisons test. ***p < 0.001, ****p < 0.0001.

(C) Schematic illustration of experimental design of AAV1 cell-internalization assay. Purified AAV1 vector was added to parental HEK293-EB cells or independent AAVR-KO clones #1 and #11. After 1 h of incubation, cells were washed with PBS and subsequent treatment with trypsin was performed to remove surface-bound AAV1 particles. Cells were harvested and subjected to genomic DNA extraction. AAV1 vector genomes were quantified by qPCR, normalized to Actb genomic sequence, and expressed relative to parental HEK293-EB cells.

(D) Both AAVR-KO clones showed significantly reduced trypsin-resistant cell-internalized AAV1 vector genomes compared with parental cells, supporting reduced AAVR-dependent uptake or cell-associated retention. Data are presented as mean ± SD. Relative expression was calculated using the 2^{-ddCt} method. Statistical analysis was performed on dCt values using ordinary one-way ANOVA followed by Dunnett's multiple comparison test. *p < 0.05.

extracellular degradation as the sole explanation for the loss of supernatant VG and support AAVR-dependent cellular uptake or retention of extracellular AAV1 particles. To examine whether this effect was serotype dependent, we performed the same assay using AAV4, which is considered relatively independent of AAVR.[12] In contrast to AAV1, AAV4 showed no significant difference in supernatant VG levels between parental HEK293-EB and AAVR-KO cells over time (Figure S3), supporting the specificity of the AAVR-dependent phenotype. We further assessed short-term AAV vector internalization by quantifying trypsin-resistant cell-associated AAV1 genomes after 1 h of exposure. Purified AAV1 vector was added to parental HEK293-EB cells or independent AAVR-KO clones #1 and #11. After 1 h of incubation, cells were washed with PBS and subsequent treatment with trypsin was performed to remove surface-bound AAV1 particles. Cells were harvested and subjected to genomic DNA extraction. AAV1 vector genomes were quantified by qPCR, normalized to Actb genomic sequence, and expressed relative to parental HEK293-EB cells. AAVR-KO clones showed significantly reduced trypsin-resistant AAV1 VG levels compared with parental HEK293-EB cells (Figures 2C and 2D). Together, these findings indicate that AAVR contributes to the cellular uptake, retention, or removal of extracellular AAV particles from the culture medium in HEK293-EB cells. In the context of active vector production, this process may contribute

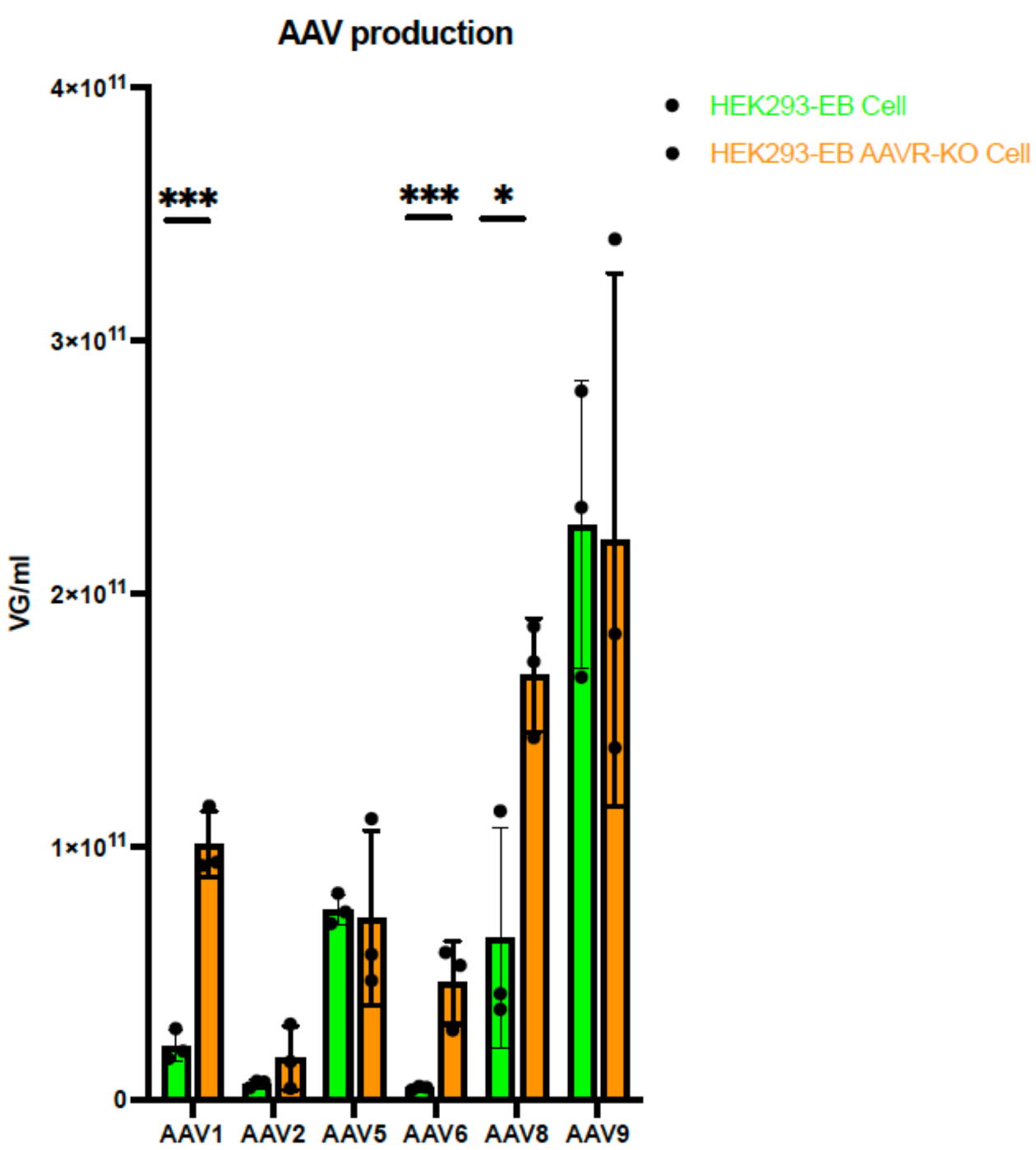

Figure 3. AAVR ablation enhances extracellular recovery of selected AAV serotypes

Supernatant VG titers following production of AAV1, AAV2, AAV5, AAV6, AAV8, and AAV9 in parental HEK293-EB or AAVR-KO clone #1 cells. AAVR knockout significantly increased extracellular titers of AAV1, AAV6, and AAV8, whereas AAV2, AAV5, and AAV9 showed no significant change. Data represent mean ± SD. Data are presented as mean ± SD (n = 3 technical replicates within a single experiment). Statistical analysis was performed on log10-transformed data using two-way ANOVA with Sidak's multiple comparisons test. *$p < 0.05$, ***$p < 0.001$.

to the recapture of released AAV particles and thereby influence supernatant VG accumulation.

We then asked whether reduced cellular uptake translates into improved vector recovery during active production. Multiple AAV serotypes (AAV1, 2, 5, 6, 8, and 9) were produced in parental and AAVR-KO cells, and supernatant VG titers were quantified (Figure 3). AAVR knockout significantly increased extracellular titers of AAV1, AAV6, and AAV8, whereas AAV5 and AAV9 showed no significant improvement. Notably, a significant interaction between cell type and serotype was observed (two-way ANOVA, interaction term $p = 0.0003$), indicating that the effect of AAVR ablation on supernatant VG titers differed among serotypes (Table S2). To assess whether this production phenotype was reproduced in other AAVR-KO clones, we produced AAV1 in an AAVR-heterozygous clone (#4) and independent AAVR-KO clones (#11 and #17). Among these clones, AAVR-KO clone #11 showed a significant increase in supernatant AAV1 VG titer compared with parental HEK293-EB cells, whereas AAVR-heterozygous clone #4 and AAVR-KO clone #17 did not show significant increases (Figure S4). These findings suggest that AAVR disruption can enhance supernatant AAV1 recovery, but that the magnitude of this effect varies among independently isolated clones and does not fully exceed clone-to-clone variability.

To further examine whether AAVR expression levels influence extracellular vector genome recovery during production, we performed a complementary gain-of-function experiment by overexpressing AAVR in HEK293-EB cells. Cells were transfected with pR2C1, pHelper, pAAV-ZsGreen1, and either the pCAX empty vector backbone or pCAX-CAG-AAVR at the same 1:1:1:1 molar ratio. Vector genomes in the culture supernatant and cell pellet were quantified at 3 and 6 days post-transfection. AAVR overexpression reduced the accumulation of extracellular vector genomes from day 3 to day 6 (Figure S5). Unexpectedly, AAVR overexpression also reduced cell-pellet vector genomes at day 3, suggesting that AAVR expression may influence vector production or intracellular vector handling in addition to extracellular particle re-uptake. Together, these loss- and gain-of-function data support the involvement of AAVR expression levels in supernatant vector recovery, while also indicating that AAVR may affect AAV production through mechanisms beyond simple re-uptake.

Together, these results indicate that AAVR contributes to the cellular re-uptake or retention of extracellular AAV particles in HEK293-EB producer cells, extending its functional relevance from viral entry to vector recovery during production.

DISCUSSION

In this study, we identify AAVR as a previously underrecognized constraint on extracellular AAV vector recovery during production. While AAVR has been extensively characterized as a critical entry factor for multiple AAV serotypes,[10,13,14] our findings extend this role to the production aspect, where the same receptor-mediated interaction can affect the amount of vector remaining in the culture supernatant. Genetic ablation of AAVR reduced cellular uptake of AAV particles and enhanced extracellular titers in a serotype-dependent manner. These results support the idea that producer cells can recapture extracellular AAV particles through an AAVR-dependent process, thereby limiting supernatant-based vector recovery.

Our data support a model in which AAVR-dependent cellular uptake or retention contributes to the substantial loss of extracellular AAV vector genomes from the culture supernatant during vector production. In the AAV1 medium-retention assay, the difference in supernatant AAV1 VG levels between parental HEK293-EB and AAVR-KO cells increased over time, consistent with cumulative cellular uptake or retention in AAVR-expressing cells rather than a simple transient adsorption effect (Figure 2B). In addition, the decline in log10-transformed supernatant AAV1 VG levels was steeper in parental HEK293-EB cells than in AAVR-KO clones over the 9-day observation period (HEK293-EB cells: −0.1008 log10 VG/day, AAVR-KO clones #1: −0.0564 log10 VG/day, AAVR-KO clones #11: −0.0558 log10 VG/day, and AAVR-KO clones #17: −0.0422 log10 VG/day). This difference in the rate of VG loss argues against

uniform extracellular degradation as the sole explanation for the decline in supernatant VG and supports a contribution of AAVR-dependent cellular uptake or retention. The short-term internalization assay further showed that AAVR-KO clones contained lower levels of trypsin-resistant cell-associated AAV1 genomes after 1 h of exposure, supporting reduced AAVR-dependent internalization or cell association (Figures 2C and 2D). In contrast, AAV4, a relatively AAVR-independent serotype, showed no clear difference in supernatant VG retention between parental HEK293-EB cells and AAVR-KO cells (Figure S3). Together, these findings support a contribution of AAVR to the cellular recapture of extracellular AAV particles.

The complementary AAVR overexpression experiment further supports the involvement of AAVR expression levels in vector genome recovery from supernatant, while also suggesting additional complexity. AAVR overexpression reduced the accumulation of extracellular vector genomes between day 3 and day 6, consistent with a role for AAVR in limiting supernatant recovery (Figure S5). Unexpectedly, AAVR overexpression also reduced cell-associated vector genomes at an early production time point (Figure S5). This observation suggests that AAVR may influence vector production, intracellular trafficking, or intracellular vector stability through mechanisms beyond simple uptake or retention of extracellular particles. Although the underlying mechanism remains to be determined, these data emphasize that AAVR expression may affect AAV vector production biology in more than one way.

From a manufacturing perspective, our findings suggest that supernatant vector recovery is influenced not only by intracellular assembly and release efficiency but also by post-release cellular interactions. Traditional optimization strategies have focused on improving transfection efficiency, capsid assembly, or secretion. Our study introduces receptor ablation as a complementary and conceptually distinct strategy, preventing vector loss rather than enhancing production *per se*. This approach may be particularly valuable for serotypes with strong AAVR dependency and high extracellular release capacity. It is also conceivable that AAV capsid assembly is not entirely uniform, resulting in particle-to-particle variability in infectivity.[15] If receptor-mediated re-uptake preferentially captures particles with higher entry competence, AAVR-dependent internalization could act as a selective filter within producer cultures. Under this scenario, vectors generated in AAVR-deficient cells may not only accumulate extracellularly but could also exhibit altered infectivity profiles. Although speculative, this possibility warrants further investigation.

Several limitations warrant consideration. Our study was conducted in HEK293-derived producer cells, and the impact of AAVR ablation in alternative manufacturing platforms remains to be determined. Although extracellular titers increased for specific serotypes, potential effects on vector quality, infectivity, and capsid integrity require further evaluation. Future studies examining scalability, long-term stability of AAVR-KO lines, and compatibility with suspension-based production systems will be important to assess the broader applicability of this approach.

In addition to AAVR-mediated uptake, cell-surface attachment factors may also contribute to the retention or recapture of extracellular AAV particles. This possibility may be particularly relevant for AAV2, which binds heparan sulfate proteoglycans (HSPG) as a primary attachment factor.[16] HSPG-mediated re-binding to producer cells could limit the amount of AAV2 remaining in the culture supernatant and may partly explain why AAVR disruption alone did not clearly increase extracellular AAV2 recovery in our study. More broadly, recent identification of AAVR2/carboxypeptidase D (CPD) as an alternate AAV receptor for selected AAV clades,[17] including AAV8-related vectors, indicates that AAV cell entry and cell-surface attachment are mediated by multiple serotype-dependent factors. It should also be noted that AAVR dependence may differ between particle internalization and productive transduction. Recent studies have suggested that AAV particles can still be internalized in certain AAVR-deficient cellular contexts, whereas AAVR remains important for productive intracellular trafficking and transgene expression.[18] Consistent with this complexity, our short-term internalization assay showed that AAVR disruption reduced, but did not necessarily eliminate, trypsin-resistant cell-associated AAV1 genomes in HEK293-EB cells (Figures 2C and 2D). Recent CRISPR-based screening in HEK293 producer cells further supports the idea that host factors associated with AAV infection, attachment, and vesicular trafficking can also influence AAV vector production biology.[19] This suggests that cell-surface and intracellular pathways may affect AAV vector production and supernatant VG output through mechanisms beyond viral entry alone. In addition, AAVR overexpression reduced the accumulation of extracellular AAV1 vector genomes in the culture supernatant from day 3 to day 6 during production (Figure S5), further supporting the involvement of AAVR expression levels in supernatant vector recovery. However, because AAVR overexpression also reduced cell-associated vector genomes at day 3, these data suggest that AAVR may influence vector production or intracellular vector handling in addition to extracellular particle re-uptake. Thus, AAVR-mediated cellular uptake or retention likely represents only one component of a broader network of producer-cell surface and intracellular processes that can influence supernatant-based vector recovery. Future producer-cell engineering strategies may therefore benefit from considering serotype-specific receptors and attachment factors, including glycans and other AAV-interacting surface proteins, in addition to AAVR.

In summary, our study identifies AAVR-mediated cellular uptake or retention as a modifiable post-release process that can influence supernatant-based AAV vector recovery. Although AAVR disruption is not universally sufficient to enhance production across all clones or serotypes, it reveals an additional layer of producer-cell biology that affects extracellular vector accumulation. These findings provide a rationale for considering receptor-mediated particle recapture in

future producer-cell engineering strategies aimed at improving supernatant-based AAV vector recovery.

MATERIALS AND METHODS

Cell culture

HEK293-EB cells (HEK293 cells stably expressing the E1 gene region and BCL-XL)[11] were cultured at 37°C in a 5% CO_2 atmosphere in Dulbecco's modified Eagle medium (DMEM, Sigma-Aldrich, St. Louis, MO). The medium was supplemented with 10% (v/v) fetal bovine serum (Biowest, Nuaillé, France), penicillin, and streptomycin (Sigma-Aldrich). AAVR-KO cells were cultured under the same conditions.

Plasmid construction

Guide RNAs (gRNAs; 20-bp target sequences) targeting the KIAA0319L gene (GenBank: NM_024874.5) were designed using the CRISPRdirect web tool (https://crispr.dbcls.jp/).[20] DNA oligonucleotides were synthesized by Eurofins Genomics Japan (https://eurofinsgenomics.jp/jp/home/). The gRNA insert was generated by PCR using the designed primer set and cloned into an AflII-digested gRNA backbone vector.[21,22]

Preparation of purified AAV vectors for transduction and medium-retention assay

HEK293-EB cells were cultured for 3 days until reaching approximately 90% confluence. Cells were then co-transfected with the appropriate Rep/Cap plasmid, pAAV-ZsGreen1, and pHelper at a plasmid ratio of 1:1:2 using PEI Max under serum-free conditions. Culture supernatants were harvested 10 days after transfection, clarified by centrifugation at 10,000 × g for 10 min, and filtered through a 0.45-μm filter. AAV vectors were purified using affinity AVR gel (Tosoh) as previously described,[23] with minor modifications. AVR gel was packed into a Tricorn empty column (Cytiva), and the column was connected to an ÄKTA avant 25 chromatography system (Cytiva). The column was equilibrated with wash buffer consisting of 20 mM Tris-HCl, pH 7.4, 500 mM NaCl, and 0.5 mM $CaCl_2$ before sample loading. After sample application, the column was washed with 20 column volumes of wash buffer to remove impurities. Bound AAV particles were eluted with elution buffer consisting of 20 mM Tris-HCl, pH 7.4, and 2 M $MgCl_2$. The eluate was filtered through a 0.22-μm filter and concentrated and buffer-exchanged into PBS using a 100-kDa Amicon Ultra-15 centrifugal filter unit (Merck). Vector genome titers of the purified AAV preparations were quantified using the AAVpro Titration Kit (for real-time PCR) Ver.2 (Takara Bio) on a QuantStudio 4 Real-Time PCR System (Thermo Fisher Scientific).

AAV transduction assay

HEK293-EB or AAVR-KO cells were seeded in 6-well culture plates at the same density used for AAV vector production and cultured at 37°C in a 5% CO_2 atmosphere until they reached subconfluence. Cells were then transduced with ZsGreen1-expressing AAV vectors. For the analysis of AAV1 transduction across independent AAVR-KO clones, parental HEK293-EB cells and AAVR-KO clones #1, #11, and #17 were transduced with AAV1-ZsGreen1 (5×10^{10} VG/well). For the serotype-dependent transduction assay, parental HEK293-EB cells and AAVR-KO clone #1 cells were transduced with AAV1, AAV2, AAV5, AAV6, AAV8, or AAV9 vectors expressing ZsGreen1. The input doses were as follows: AAV1, 6.1×10^4 VG/cell; AAV2, 1.9×10^4 VG/cell; AAV5, 5.2×10^4 VG/cell; AAV6, 1.5×10^4 VG/cell; AAV8, 1.2×10^5 VG/cell; and AAV9, 2.9×10^4 VG/cell. ZsGreen1 fluorescence images were acquired using an ECLIPSE Ts2 microscope (Nikon). Transduction efficiency was evaluated by ZsGreen1 expression and quantified as the percentage of ZsGreen1-positive cells using a BD FACS Melody cell sorter.

AAV medium-retention assay

HEK293-EB or AAVR-KO cells were seeded in 6-well culture plates at the same density used for AAV vector production and cultured at 37°C in a 5% CO_2 atmosphere until they reached subconfluence. Purified AAV vectors were then added directly to the culture medium. For the AAV1 medium-retention assay, AAV1-ZsGreen1 was added at 5×10^{10} VG/well in 2 mL of medium. Culture supernatants were collected at 3, 6, and 9 days after vector addition. For the AAV4 medium-retention assay, AAV4-ZsGreen1 was added at 2.1×10^{10} VG/well in 2 mL of medium, and culture supernatants were collected at 3, 7, and 10 days after vector addition. Cells were maintained without medium exchange during the assay. Vector genomes remaining in the culture supernatant were quantified by qPCR.

Supernatant AAV vector recovery assay

HEK293-EB or AAVR-KO cells were seeded in 6-well culture plates at a density of 4×10^4 cells/cm^2 and cultured for 3 days at 37°C in a 5% CO_2 atmosphere until reaching approximately 90% confluence. Cells were then transfected with pRep/Cap plasmids (AAV1: Addgene plasmid #112862; AAV2: Addgene plasmid #104963; AAV4: pSV40oriAAV4-2, AAV5: Addgene plasmid #104964; AAV6: Addgene plasmid #110660; AAV8: Addgene plasmid #112864; AAV9: Addgene plasmid #112865), pGOI (pZsGreen1; Takara Bio), and pHelper (Addgene plasmid #112867) at a mass ratio of 1:1:2. Transfections were performed in serum-free DMEM supplemented with 1% penicillin-streptomycin and 1% GlutaMAX-I (Thermo Fisher Scientific, Waltham, MA, USA) using polyethylenimine (PEI Max; Polysciences, Warrington, PA, USA) at a DNA:PEI Max ratio of 1:4. AAV vectors were harvested from the culture supernatant 264 h post-transfection, clarified by centrifugation at 10,000 × g for 15 min at 4°C, and filtered through a 0.45-μm filter (Thermo Fisher Scientific).

Vector genome quantification by qPCR

AAV vector genome titers were quantified using the AAVpro Titration Kit (for Real-Time PCR) Ver.2 (Takara Bio, Japan) according to the manufacturer's instructions. Briefly, samples were treated with DNase I to remove non-encapsidated DNA, followed by heat inactivation and lysis to release encapsidated vector genomes. The extracted vector genome samples were diluted and subjected to real-time qPCR using the AAV titer primer set and positive-control standard DNA provided in the kit. qPCR was performed on a

QuantStudio 4 Real-Time PCR System (Thermo Fisher Scientific). The cycling conditions were as follows: 95°C for 2 min, followed by 35 cycles of 95°C for 5 s and 60°C for 30 s. Vector genome copy numbers were calculated from the standard curve and expressed as vector genomes per milliliter of culture supernatant or as vector genomes per cell-associated fraction, as appropriate.

Western blotting

Proteins were separated on 3%–8% gradient gels (Invitrogen; supplied with running buffer) and transferred onto 0.45 μm PVDF membranes (Invitrogen) using a semi-dry transfer system (Trans-Blot Turbo, Bio-Rad). Tri-Glycine transfer buffer (Novex, Invitrogen) supplemented with 10% methanol was used for protein transfer. The membranes were incubated overnight at 4°C with primary antibodies diluted as follows: anti-AAVR antibody (ab315027, Abcam; 1:500) and anti-β-actin antibody (#3700S, Cell Signaling Technology; 1:3,000). After washing, membranes were incubated with HRP-conjugated secondary antibodies for 1 h at room temperature: anti-mouse IgG (NA9310-1ML; 1:10,000) and anti-rabbit IgG (1:5,000).

AAV vector cell internalization assay

AAV vector cell internalization assay was performed as follows.[24] Purified AAV vector was added to cells and incubated for 1 h at 37°C. Cells were washed with PBS and subsequently treated with trypsin at 37°C for 5 min to remove surface-bound viral particles. The cells were then harvested by centrifugation, and genomic DNA was extracted with DNeasy Blood and Tissue Kits (QIAGEN). AAV vector genomes were quantified by qPCR (F primer:gtgtacaaggccaagtccgt, R primer:ccacttctggttcttggcgt) and normalized to Actb (F primer:gggacctgactgactacctcatg, R primer:agcttctccttaatgtcacgcac) as an internal genomic control. Relative trypsin-resistant cell-associated AAV vector genome levels were calculated using the ddCt method, with parental HEK293-EB cells.

Statistical analysis

Statistical analyses were performed using GraphPad Prism 10. Data are presented as mean ± SD. The number of replicates and whether they represent technical or biological replicates are indicated in each figure legend. For experiments comparing multiple serotypes in the same assay, vector genome titers were log10-transformed before statistical analysis.

DATA AND CODE AVAILABILITY

All data supporting the findings of this study are included in the article and its supplemental information. Additional data are available from the corresponding author upon reasonable request.

ACKNOWLEDGMENTS

We gratefully acknowledge Tosoh Corporation for kindly providing the AVR gel. pDGM6 was a gift from David Russell (Addgene plasmid # 110660; http://n2t.net/addgene:110660),[25] pR2C1, pR2C2, pR2C5, pR2C8, pR2C9, and pAdDeltaF6 was a gift from James M. Wilson (Addgene plasmid # 112862, # 104963, # 104964, # 110660, # 112864, # 112865, #112867), pSV40oriAAV4-2 was a kind gift from ROBERT M. KOTIN.[26] This research was supported by AMED under grant number JP23ae0201001, JP23ae0201005, JP25bm1523001, JP25bm1523007, JP243fa827003, JP253fa627001, JP24bk0304009, and JSPS KAKENHI grant number JP24H00646, JP25K02260.

AUTHOR CONTRIBUTIONS

Y.T. conceived the idea, designed all the experiments, and wrote the manuscript. K.S., K.Y., N.A.G.M., Y.K., and A.O. performed the experiments, and T.O. supervised the study.

DECLARATION OF INTERESTS

K.Y. is an employee of Tosoh Corporation. The laboratory has received research funding from Tosoh Corporation under a collaborative research agreement unrelated to the work reported in this manuscript. The funding company had no role in the conception, design, conduct, analysis, or interpretation of the present study, or in the decision to submit the manuscript for publication. All other authors declare no competing interests.

DECLARATION OF GENERATIVE AI AND AI-ASSISTED TECHNOLOGIES IN THE WRITING PROCESS

During the preparation of this work, the authors used ChatGPT for English language editing and improvement of readability. The authors reviewed and edited the output as needed and take full responsibility for the content of the published article.

SUPPLEMENTAL INFORMATION

Supplemental information can be found online at https://doi.org/10.1016/j.omta.2026.201830.

REFERENCES

1. Podsakoff, G., Wong, K.K., Jr., and Chatterjee, S. (1994). Efficient gene transfer into nondividing cells by adeno-associated virus-based vectors. J. Virol. *68*, 5656–5666. https://doi.org/10.1128/JVI.68.9.5656-5666.1994.
2. Ellis, B.L., Hirsch, M.L., Barker, J.C., Connelly, J.P., Steininger, R.J., 3rd, and Porteus, M.H. (2013). A survey of ex vivo/in vitro transduction efficiency of mammalian primary cells and cell lines with Nine natural adeno-associated virus (AAV1-9) and one engineered adeno-associated virus serotype. Virol. J. *10*, 74. https://doi.org/10.1186/1743-422X-10-74.
3. Vandenberghe, L.H., Xiao, R., Lock, M., Lin, J., Korn, M., and Wilson, J.M. (2010). Efficient serotype-dependent release of functional vector into the culture medium during adeno-associated virus manufacturing. Hum. Gene Ther. *21*, 1251–1257. https://doi.org/10.1089/hum.2010.107.
4. Kurosawa, Y., Tsunekawa, Y., Wada, M., Aizen, Y., Nitahara-Kasahara, Y., and Okada, T. (2024). Purification of Adeno-Associated Viral Vector Serotype 9 Using Ceramic Hydroxyapatite Chromatography and its Analysis. Curr. Protoc. *4*, e1068. https://doi.org/10.1002/cpz1.1068.
5. Miyaoka, R., Tsunekawa, Y., Kurosawa, Y., Sasaki, T., Onodera, A., Sakamoto, K., Kakiuchi, Y., Wada, M., Nitahara-Kasahara, Y., Hayashita-Kinoh, H., and Okada, T. (2023). Development of a novel purification method for AAV vectors using tangential flow filtration. Biotechnol. Bioeng. *120*, 3311–3321. https://doi.org/10.1002/bit.28524.
6. Wada, M., Uchida, N., Posadas-Herrera, G., Hayashita-Kinoh, H., Tsunekawa, Y., Hirai, Y., and Okada, T. (2023). Large-scale purification of functional AAV particles packaging the full genome using short-term ultracentrifugation with a zonal rotor. Gene Ther. *30*, 641–648. https://doi.org/10.1038/s41434-023-00398-x.
7. Chaubal, A.S., Horax, R., Yehl, C.J., Wickramasinghe, S.R., Qian, X., Wang, L., and Zydney, A.L. (2026). Downstream Process Intensification for AAV Purification by Affinity Chromatography Using Single Pass Tangential Flow Filtration. Biotechnol. Bioeng. *123*, 174–185. https://doi.org/10.1002/bit.70090.
8. Khaparde, A., Patra, R., Roy, S., Babu G R, S., and Ghosh, A. (2025). Improving AAV Production Yield and Quality for Different Serotypes Using Distinct Processing Methods. ACS Omega *10*, 22657–22670. https://doi.org/10.1021/acsomega.4c10900.
9. Konno, A., and Hirai, H. (2020). Efficient whole brain transduction by systemic infusion of minimally purified AAV-PHP.eB. J. Neurosci. Methods *346*, 108914. https://doi.org/10.1016/j.jneumeth.2020.108914.

10. Pillay, S., Meyer, N.L., Puschnik, A.S., Davulcu, O., Diep, J., Ishikawa, Y., Jae, L.T., Wosen, J.E., Nagamine, C.M., Chapman, M.S., and Carette, J.E. (2016). An essential receptor for adeno-associated virus infection. Nature *530*, 108–112. https://doi.org/10.1038/nature16465.

11. Tomono, T., Hirai, Y., Okada, H., Miyagawa, Y., Adachi, K., Sakamoto, S., Kawano, Y., Chono, H., Mineno, J., Ishii, A., et al. (2018). Highly Efficient Ultracentrifugation-free Chromatographic Purification of Recombinant AAV Serotype 9. Mol. Ther. Methods Clin. Dev. *11*, 180–190. https://doi.org/10.1016/j.omtm.2018.10.015.

12. Dudek, A.M., Pillay, S., Puschnik, A.S., Nagamine, C.M., Cheng, F., Qiu, J., Carette, J.E., and Vandenberghe, L.H. (2018). An Alternate Route for Adeno-associated Virus (AAV) Entry Independent of AAV Receptor. J. Virol. *92*, e02213. https://doi.org/10.1128/JVI.02213-17.

13. Meyer, N.L., and Chapman, M.S. (2022). Adeno-associated virus (AAV) cell entry: structural insights. Trends Microbiol. *30*, 432–451. https://doi.org/10.1016/j.tim.2021.09.005.

14. Pillay, S., Zou, W., Cheng, F., Puschnik, A.S., Meyer, N.L., Ganaie, S.S., Deng, X., Wosen, J.E., Davulcu, O., Yan, Z., et al. (2017). Adeno-associated Virus (AAV) Serotypes Have Distinctive Interactions with Domains of the Cellular AAV Receptor. J. Virol. *91*, e00391. https://doi.org/10.1128/JVI.00391-17.

15. Wörner, T.P., Bennett, A., Habka, S., Snijder, J., Friese, O., Powers, T., Agbandje-McKenna, M., and Heck, A.J.R. (2021). Adeno-associated virus capsid assembly is divergent and stochastic. Nat. Commun. *12*, 1642. https://doi.org/10.1038/s41467-021-21935-5.

16. Kern, A., Schmidt, K., Leder, C., Müller, O.J., Wobus, C.E., Bettinger, K., Von der Lieth, C.W., King, J.A., and Kleinschmidt, J.A. (2003). Identification of a heparin-binding motif on adeno-associated virus type 2 capsids. J. Virol. *77*, 11072–11081. https://doi.org/10.1128/jvi.77.20.11072-11081.2003.

17. Dhungel, B.P., Xu, H., Nagarajah, R., Vitale, J., Wong, A.C.H., Gokal, D., Feng, Y., Tabar, M.S., Metierre, C., Parsania, C., et al. (2025). An alternate receptor for adeno-associated viruses. Cell *188*, 4924–4935.e23. https://doi.org/10.1016/j.cell.2025.06.026.

18. Hao, S., Zhang, X., Ning, K., Feng, Z., Park, S.Y., Aksu Kuz, C., McFarlin, S., Richart, D., Cheng, F., Zhang, E.Y., et al. (2023). Identification of host essential factors for recombinant AAV transduction of the polarized human airway epithelium. J. Virol. *97*, e0133023. https://doi.org/10.1128/jvi.01330-23.

19. O'Driscoll, E.E., Arora, S., Lang, J.F., Davidson, B.L., and Shalem, O. (2025). CRISPR screen reveals modifiers of rAAV production including known rAAV infection genes playing an unexpected role in vector production. Mol. Ther. Methods Clin. Dev. *33*, 101408. https://doi.org/10.1016/j.omtm.2025.101408.

20. Naito, Y., Hino, K., Bono, H., and Ui-Tei, K. (2015). CRISPRdirect: software for designing CRISPR/Cas guide RNA with reduced off-target sites. Bioinformatics *31*, 1120–1123. https://doi.org/10.1093/bioinformatics/btu743.

21. Suzuki, K., Tsunekawa, Y., Hernandez-Benitez, R., Wu, J., Zhu, J., Kim, E.J., Hatanaka, F., Yamamoto, M., Araoka, T., Li, Z., et al. (2016). In vivo genome editing via CRISPR/Cas9 mediated homology-independent targeted integration. Nature *540*, 144–149. https://doi.org/10.1038/nature20565.

22. Tsunekawa, Y., Terhune, R.K., Fujita, I., Shitamukai, A., Suetsugu, T., and Matsuzaki, F. (2016). Developing a de novo targeted knock-in method based on in utero electroporation into the mammalian brain. Development *143*, 3216–3222. https://doi.org/10.1242/dev.136325.

23. Yoshida, K., Tsunekawa, Y., Kurihara, K., Watanabe, K., Makino-Manabe, Y., Wada, M., Tanaka, T., Ide, T., and Okada, T. (2023). Engineering a highly durable adeno-associated virus receptor for analytical applications. Mol. Ther. Methods Clin. Dev. *31*, 101157. https://doi.org/10.1016/j.omtm.2023.101157.

24. Berry, G.E., and Tse, L.V. (2017). Virus Binding and Internalization Assay for Adeno-associated Virus. Bio. Protoc. *7*, e2110. https://doi.org/10.21769/BioProtoc.2110.

25. Gregorevic, P., Blankinship, M.J., Allen, J.M., Crawford, R.W., Meuse, L., Miller, D.G., Russell, D.W., and Chamberlain, J.S. (2004). Systemic delivery of genes to striated muscles using adeno-associated viral vectors. Nat. Med. *10*, 828–834. https://doi.org/10.1038/nm1085.

26. Chiorini, J.A., Yang, L., Liu, Y., Safer, B., and Kotin, R.M. (1997). Cloning of adeno-associated virus type 4 (aav4) and generation of recombinant aav4 particles. J. Virol. *71*, 6823–6833. https://doi.org/10.1128/JVI.71.9.6823-6833.1997.

OMTA, Volume *34*

Supplemental information

AAVR disruption as a producer-cell engineering strategy for enhanced supernatant-based recovery of selected AAV serotypes

Kenji Sakamoto, Kouhei Yoshida, Yuji Tsunekawa, Naymel A. Guzman Mendoza, Yui Komaki, Azusa Onodera, and Takashi Okada

A

	Position	Targeting sequence (coding KIAA0319L)
gRNA 1	Chromosome 1: 35,555,095 - 35,555,120	ATTCAACAGATACTTACGTC AGG
gRNA 2	Chromosome 1: 35,553,781 - 35,553,806	CCTGGCAAGAGGCTTTACCC TGG

Genotyping primer	Position	Sequence
Amp F primer	Chromosome 1: 35,553,450 – 35,553,479 (+)	AACCCCAAAGCTGATGTTACTGAAATTGCT
Amp R primer	Chromosome 1: 35,556,038 – 35,556,067 (-)	TGGTGATGTGTTGTTGTTCTTGCGTTTGGT

B

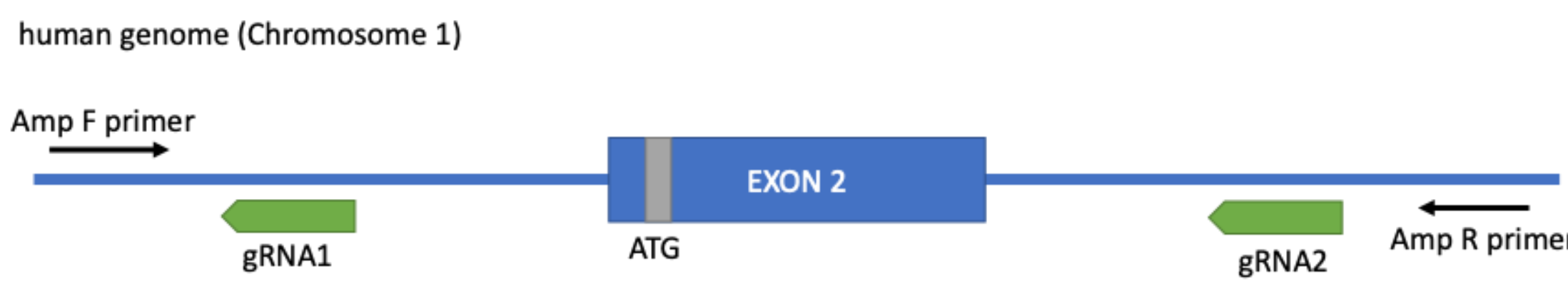

Figure S1: CRISPR/Cas9 targeting strategy for AAVR (KIAA0319L) knockout
(A) gRNA and genotyping primer sequences. Genomic target sites for CRISPR/Cas9-mediated disruption of AAVR (KIAA0319L) are shown. Two guide RNAs (gRNA1 and gRNA2) flanking exon 2 were designed, and their genomic positions and targeting sequences are listed. Genotyping primers flanking the targeted region were used to detect exon 2 deletion; their genomic positions and sequences are provided.
(B) Schematic illustration of the AAVR exon 2 targeting strategy. The positions of gRNA target sites and genotyping primers relative to exon 2 are illustrated.

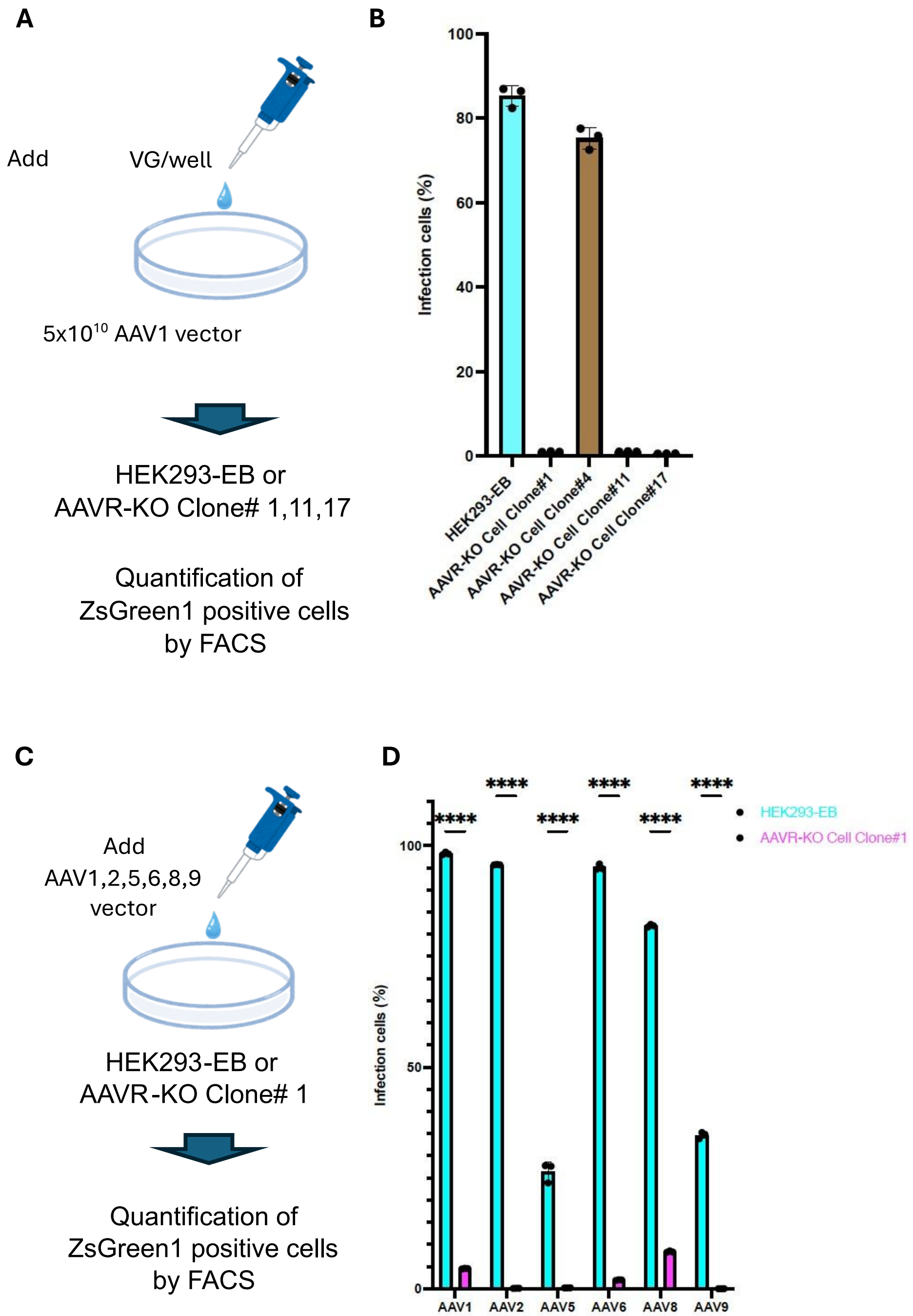

Figure S2: Quantitative analysis of serotype-dependent AAV transduction in AAVR-knockout cells.

(A) Schematic illustration of experimental design for assessing transduction efficiency of AAV1 across parental HEK293-EB and independent AAVR-KO clones (#1, #11, #17).

(B) Transduction efficiency of AAV1 across parental HEK293-EB and AAVR-KO clones (#1, #11, #17), quantified by ZsGreen1 expression. Data represent mean ± SD. Data are presented as mean ± SD (n = 3 technical replicates within a single experiment).

(C) Schematic illustration of experimental design for assessing transduction efficiency of serotype-dependent transduction efficiency (AAV1, 2, 5, 6, 8, 9), corresponding to Fig. 1C.

(D) Quantification of serotype-dependent transduction efficiency corresponding to Fig. 1C. Parental HEK293-EB cells and AAVR-KO clone #1 cells were transduced with ZsGreen1-expressing AAV serotypes 1, 2, 5, 6, 8, and 9. Transduction efficiency was quantified based on the percentage of ZsGreen1-positive cells. The input doses were as follows: AAV1: 6.1×10^4 VG/cell; AAV2: 1.9×10^4 VG/cell; AAV5: 5.2×10^4 VG/cell; AAV6: 1.5×10^4 VG/cell; AAV8: 1.2×10^5 VG/cell; and AAV9: 2.9×10^4 VG/cell. Data are presented as mean ± SD (n = 3 technical replicates from a single experiment). Statistical analysis was performed using two-way ANOVA followed by Sidak's multiple comparisons test. ****$P < 0.0001$.

A

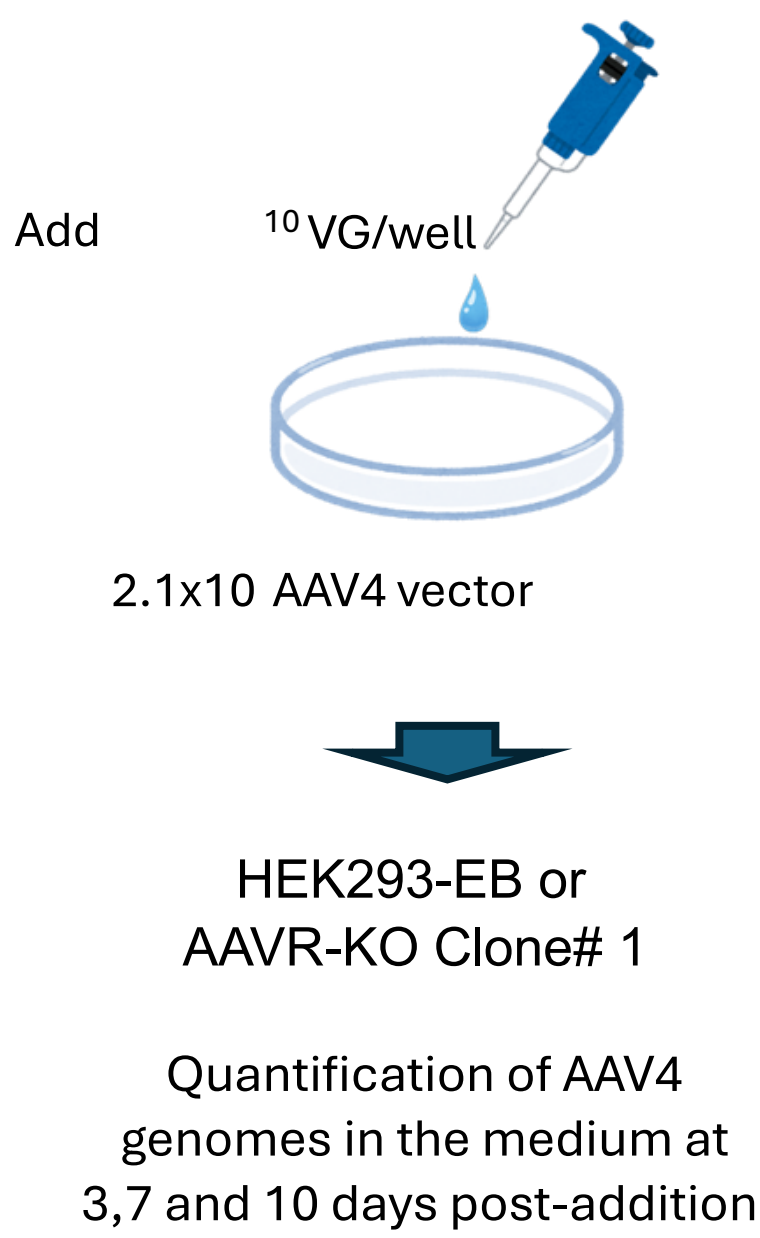

B

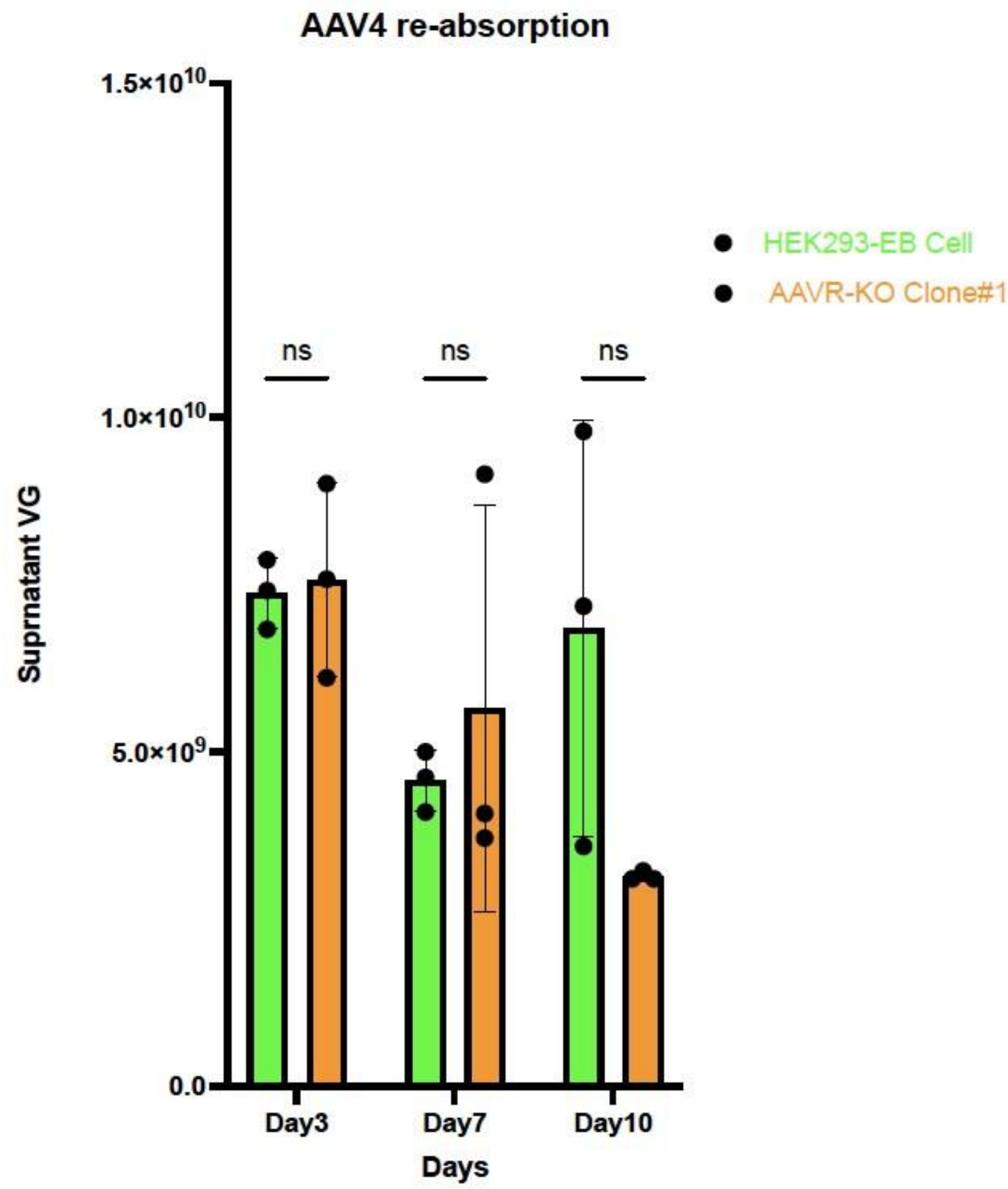

Figure S3: Cellular uptake of extracellular AAV4 particles in parental and AAVR-knockout cells.

(A) Schematic illustration of experimental design. Purified AAV4 vector (2.1×10^{10} VG per well; 1.05×10^{10} VG/mL) was added to subconfluent parental HEK293-EB or AAVR-KO clone #1 cells cultured in 6-well plates (2 mL medium per well). Vector genomes remaining in the culture supernatant were quantified at 3, 7 and 10 days post-addition.

(B) Quantification of AAV4 genomes in the supernatant. AAV4 showed no significant difference in supernatant vector genome levels between parental and AAVR-KO cells over time. Data represent mean ± SD (n = 3 technical replicates within a single experiment). Statistical analysis was performed using two -way ANOVA with Sidak's multiple comparisons test.

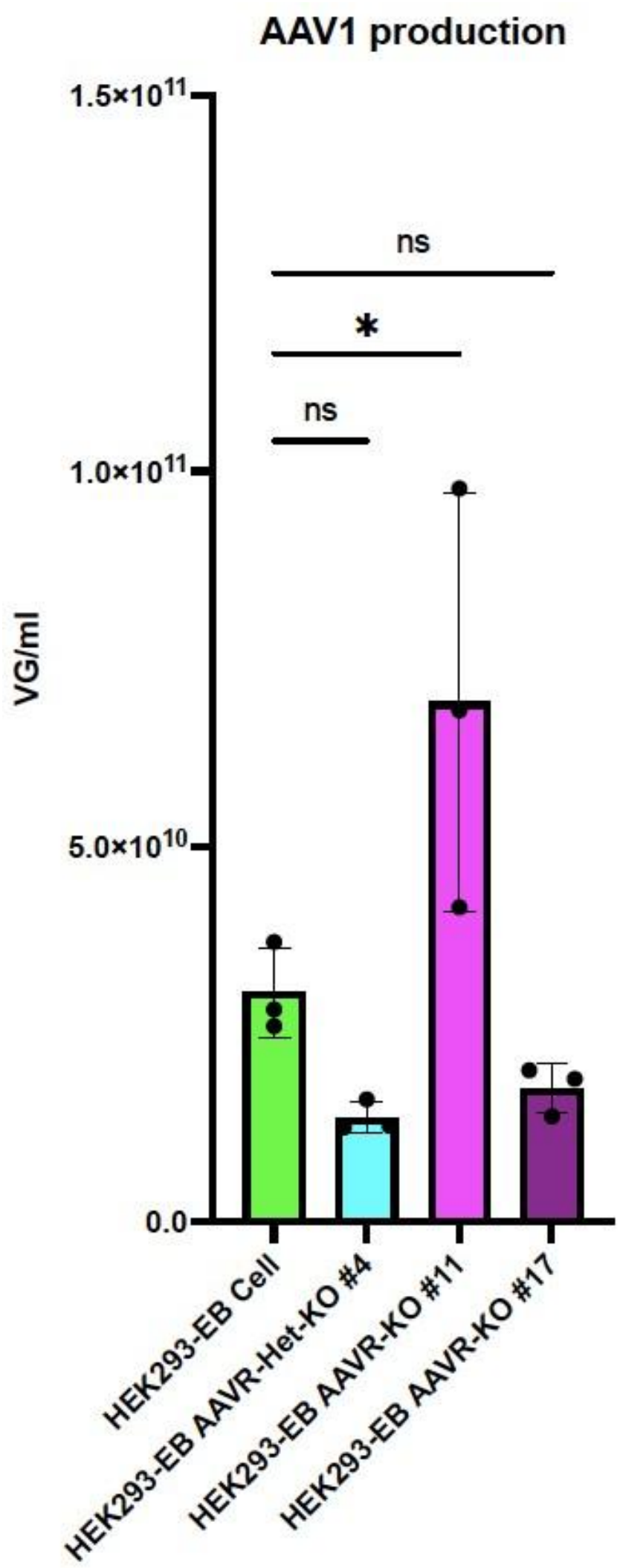

Figure S4: AAV1 extracellular recovery in AAVR-KO and HEK293-EB clones.
Supernatant VG titers following extracellular recovery of AAV1 in parental HEK293-EB or AAVR-KO clone #11, 17 and Het-KO clone#4 cells. AAVR knockout clone#11 showed significantly increased extracellular titers of AAV1but AAVR-KO clone #17 and Het-KO clone#4 showed no significant change. Data are presented as men ± SD (n = 3 technical replicates within a single experiment). Statistical analysis was performed using ordinary one-way ANOVA. *P < 0.05.

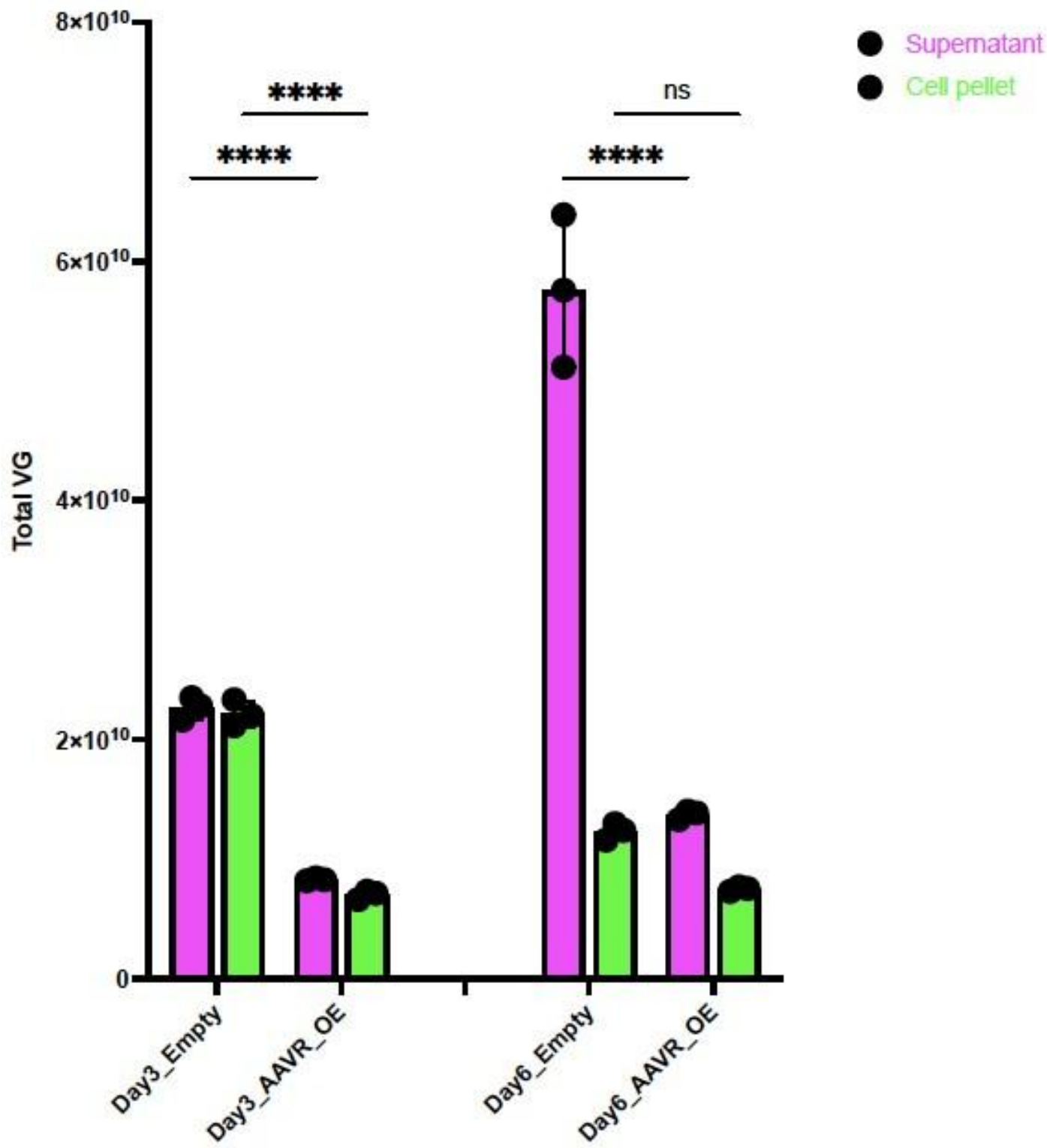

Figure S5: AAVR overexpression alters supernatant and cell-pellet AAV1 vector genome levels during production.
HEK293-EB cells were transfected with pR2C1, pHelper, pAAV-ZsGreen1, and either the pCAX empty vector backbone (Empty condition) or pCAX-CAG-AAVR (AAVR-OE condition) at a 1:1:1:1 molar ratio. AAV1 vector genomes yield from culture supernatant and cell pellet were quantified at 3 and 6 days post-transfection. AAVR overexpression reduced the accumulation of extracellular vector genomes from day 3 to day 6, consistent with the involvement of AAVR expression levels in supernatant vector recovery. AAVR overexpression also reduced cell-associated vector genomes at day 3, suggesting that AAVR may influence vector production or intracellular vector handling in addition to extracellular particle re-uptake. Data represent mean ± SD. Data are presented as mean ± SD (n = 3 technical replicates within a single experiment). Statistical analysis was performed on data using two-way ANOVA with Sidak’s multiple comparisons test. ****P < 0.0001.

Table S1. Rate of supernatant AAV1 vector genome loss in parental and AAVR-knockout HEK293-EB cells.

The rate of decline in supernatant AAV1 vector genome levels was calculated using the defined input dose as day 0. Slopes were estimated by linear regression of log10-transformed supernatant vector genome levels from day 0 to day 9.

**Cell line**	**Day0**	**Day3 mean**	**Day6 mean**	**Day9 mean**	**Linear slope, VG/day**	**log10 slope/day**	**Day9/Day0 remaining**
HEK293-EB	5.00 × 10^10	1.67 × 10^10	1.09 × 10^10	5.65 × 10^9	-4.44 * 10^9/DAY	-0.1008/DAY	11.30%
AAVR-KO #1	5.00 × 10^10	2.32 × 10^10	2.05 × 10^10	1.42 × 10^10	-3.62 * 10^9/DAY	-0.0564/DAY	28.40%
AAVR-KO #11	5.00 × 10^10	2.34 × 10^10	1.91 × 10^10	1.48 × 10^10	-3.62 * 10^9/DAY	-0.0558/DAY	29.60%
AAVR-KO #17	5.00 × 10^10	2.49 × 10^10	2.28 × 10^10	1.95 × 10^10	-3.03 * 10^9/DAY	-0.0422/DAY	39.00%

Table S2. Supernatant vector genome titers of AAV serotypes produced in parental and AAVR-knockout HEK293-EB cells.

Supernatant vector genome titers of AAV1, AAV2, AAV5, AAV6, AAV8, and AAV9 produced in parental HEK293-EB cells and AAVR-KO clone #1 cells are shown. Values are presented as log10-transformed vector genome titers. Mean differences, 95% confidence intervals, fold changes, and adjusted P values were calculated from log10-transformed data using two-way ANOVA followed by Sidak's multiple comparisons test.

Serotype	Mean log10 VG titer in parental HEK293-EB cells	Mean log10 VG titer in AAVR-KO cells	Difference, AAVR-KO − parental (log10 units)	95% CI of difference	Approximate fold change	Adjusted P value	Interpretation
AAV1	10.31	11	0.6884	0.2526 to 1.124	4.9-fold increase	0.0008	Significant increase
AAV2	9.785	10.1	0.311	−0.1248 to 0.7468	2.0-fold increase	0.2738	Not significant
AAV5	10.87	10.83	−0.04888	−0.4847 to 0.3869	0.89-fold	0.9998	Not significant
AAV6	9.655	10.64	0.9875	0.5517 to 1.423	9.7-fold increase	<0.0001	Significant increase
AAV8	10.74	11.22	0.4786	0.04278 to 0.9143	3.0-fold increase	0.0259	Significant increase
AAV9	11.35	11.31	−0.03326	−0.4690 to 0.4025	0.93-fold	>0.9999	Not significant

Supplemental Materials and Methods

AAVR knock-out cell generation

AAVR-knockout HEK293-EB clones were generated using a CRISPR/Cas9-mediated genome editing strategy. Subconfluent HEK293-EB cells were transfected with pCAX-SpCas9 and two gRNA plasmids targeting sequences flanking exon 2 of KIAA0319L using PEI-Max (Polysciences). Five days after transfection, single cells were sorted into 96-well plates using a BD FACS Melody cell sorter. After approximately 2 weeks of culture, colonies were expanded and screened by genomic PCR to identify clones carrying homozygous deletion of exon 2. Loss of AAVR protein expression was further confirmed by Western blotting.

Genotyping PCR

Genomic DNA was extracted from expanded clones using the DNeasy Blood & Tissue Kit (QIAGEN) according to the manufacturer's instructions. PCR-based genotyping was performed using PrimeSTAR GXL DNA Polymerase (Takara Bio). Each 50-µL PCR reaction contained 10 µL of 5× PrimeSTAR GXL buffer, 4 µL of dNTP mixture, 1 µL of PrimeSTAR GXL DNA Polymerase, 0.5 µL each of forward and reverse primers (10 pM), 5 µL of betaine, 1 µL of genomic DNA template (50 ng), and 28 µL of distilled water. PCR was performed using the following cycling conditions and Primer: initial denaturation at 96°C for 5 min, followed by 33 cycles of denaturation at 96°C for 20 s and extension at 68°C for 2 min (F-Primer: AACCCCAAAGCTGATGTTACTGAAATTGCT, R-Primer: TGGTGATGTGTTGTTGTTCTTGCGTTTGGT). PCR products were analyzed by electrophoresis on a 1% agarose gel, image was taken by ChemiDoc imaging system (BioRad).

AAVR overexpression production assay

HEK293-EB cells were seeded in 6-well culture plates at a density of 4 × 10^4 cells/cm^2 and cultured for 3 days at 37°C in a 5% CO_2 atmosphere until reaching approximately 90% confluence. Cells were then transfected with pR2C1, pHelper, pAAV-ZsGreen1, and either the pCAX empty vector backbone or pCAX-CAG-AAVR using PEI Max. The four plasmids were transfected at a 1:1:1:1 molar ratio. After transfection, cells were maintained in serum-free medium without medium exchange. Culture supernatants and cells were collected at 3 and 6 days post-transfection. Supernatants were clarified by

centrifugation, and cells were harvested separately as the cell-associated fraction. The harvested cells were lysed with 0.1% Triton X-100 for 2 h. Vector genomes in the culture supernatant and cell-associated fractions were quantified by qPCR.
